# Multitrophic Diversity of the Biotic Community Drives Ecosystem Multifunctionality in Alpine Grasslands

**DOI:** 10.1002/ece3.70511

**Published:** 2024-11-05

**Authors:** Hongye Su, Zhen Wang, Li Ma, Ruimin Qin, Tao Chang, Zhonghua Zhang, Junfei Yao, Xudong Li, Shan Li, Xue Hu, Jingjing Wei, Fang Yuan, Haze Adi, Zhengchen Shi, Honglin Li, Huakun Zhou

**Affiliations:** ^1^ Qinghai Provincial Key Laboratory of Restoration Ecology in Cold Regions, Northwest Institute of Plateau Biology Chinese Academy of Sciences Xining China; ^2^ College of Resources and Environment University of Chinese Academy of Sciences Beijing China; ^3^ College of Grassland Science and Technology China Agricultural University Beijing China; ^4^ College of Agriculture and Animal Husbandry Qinghai University Xining China; ^5^ College of Geographical Science Qinghai Normal University Xining China; ^6^ State Key Laboratory of Plateau Ecology and Agriculture Qinghai University Xining China

**Keywords:** biodiversity, biotic variables, community, ecosystem functions, multitrophic levels

## Abstract

Biodiversity and ecosystem multifunctionality are currently hot topics in ecological research. However, little is known about the role of multitrophic diversity in regulating various ecosystem functions, which limits our ability to predict the impact of biodiversity loss on human well‐being and ecosystem multifunctionality. In this study, multitrophic diversity was divided into three categories: plant, animal, and microbial communities (i.e., plant diversity, rodent diversity, and bacterial and fungal diversity). Also, 15 ecosystem functions were divided into four categories—water conservation, soil fertility, nutrient cycling and transformation, and community production—to evaluate the significance of biotic and abiotic variables in maintaining ecosystem multifunctionality. Results indicated that species diversity at multiple trophic levels had a greater positive impact on ecosystem multifunctionality than species diversity at a single trophic level. Notably, the specific nature of this relationship depended on the niche breadths of plants, indicating that plants played a key role in linking above and belowground trophic levels. Abiotic factors such as altitude and pH directly acted on ecosystem multifunctionality and could explain changes in ecosystem functions. Overall, our study offers valuable insights into the critical role of multitrophic species diversity in preserving ecosystem multifunctionality within alpine grassland communities, as well as strong support for the importance of biodiversity protection.

## Graphical abstract

Our results indicated that primary producers, primary consumers, and soil decomposers were crucial trophic groups for maintaining ecosystem functionality, collectively driving changes in ecosystem multifunctionality. Additionally, abiotic factors such as altitude and pH influenced ecosystem multifunctionality.

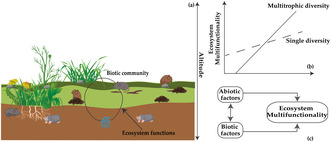

## 1 Introduction

In the past 20 years, biodiversity and ecosystem multifunctionality relationships are a central issue in both basic and applied ecology (Hooper et al. 2005; Lefcheck et al. 2015; Soliveres et al. 2016). There is clear evidence indicating that loss of biodiversity at any particular trophic level can lead to a decline in ecosystem services (e.g., production, maintenance of soil fertility, and water purification) and efficiency of resource capture (Balvanera et al. 2006; Weisser et al. 2017). Nevertheless, existing studies typically concentrated on single trophic groups, especially plant communities, overlooking the fact that biodiversity loss occurs across multiple communities (Allan et al. 2014; Antiqueira, Petchey, and Romero 2018; Wang et al. 2019). Indeed, ecosystems are complex and diverse, with food webs formed by the interactions of species at different trophic levels, thus harmonizing the ecosystem structure and functions (Seibold et al. 2018). Many experiments have shown that focusing solely on a single trophic level group may significantly underestimate the impact of biodiversity on ecosystem functioning. The underlying reason is that functional effects of different trophic groups may complement or counteract each other (Soliveres et al. 2016; Seibold et al. 2018; Luo et al. 2022; Martins et al. 2024). For example, enriching plant diversity can increase soil microbial diversity in natural ecosystems and farming systems (Garland et al. 2021), and in agricultural systems microbial diversity can also improve crop yield (Dahlstrom, McRose, and Newman 2020) and quality and facilitate the rate of nutrient acquisition by plants (Jing et al. 2015); the manipulation of plant species has knock‐on effects on other groups, such as bacteria and mycorrhiza (Hector and Bagchi 2007); an increase in plant species diversity promotes beneficial interactions between insects and plants at different trophic levels, leading to significant bottom‐up effects that influence ecosystem functions (Wan et al. 2020). Also, the interactions within multitrophic metacommunity can mediate the asynchrony and stability of communities in fluctuating environments (Firkowski et al. 2021). Currently, research on multitrophic interactions primarily focuses on the interactions among primary producers (plants), primary consumers (insects), and decomposers in the soil (bacteria, fungi, and nematodes), as well as their responses to climate change and ecosystem functioning (Granot, Belmaker, and Sandel 2019; Buzhdygan, Petermann, and Schmid 2023; Wang, Sun, Mishra, et al. 2023), with limited attention given to rodents. Rodents serve as seed consumers and dispersers, and the differences in seed characteristics directly influence their dispersal strategies. Over long‐term evolution, this has led to a mutualistic relationship between rodents and plants (Yu et al. 2014). Meanwhile, rodent digging changes soil nutrient distribution, enhances soil permeability and water absorption, and increases grassland soil surface heterogeneity, thereby indirectly impacting the plant growth environment (Laundre and Reynolds 1993). Therefore, there is an urgent need to fully understand whether changes in trophic level complexity will affect ecosystem functioning.

Studies of multiple trophic levels have relied on measuring the species diversity of habitats to uncover trophic linkages, with species at different trophic levels transferring energy and nutrients through food chains, facilitating interactions and linkages between organisms (Deraison et al. 2015; Li et al. 2020). Trophic richness will mean that a diverse range of organisms will be able to occupy suitable niches within an ecosystem, thus contributing to the maintenance of biodiversity. For a long time, ecologists have been exploring how species diversity is maintained within biological communities. The classical theory of species coexistence suggests that environmental filtering leads to species with similar functional traits occupying similar ecological niches in space, resulting in aggregated distributions (Kraft et al. 2014; Hua et al. 2024). Biological filtering mainly involves niche differentiation and fitness differences among species with similar functional traits in the community due to competition, involving both interspecific competition and intraspecific functional trait variation (Aschehoug et al. 2016).

Plants act as intermediaries connecting consumers and decomposers, providing an energy foundation for the food chain through photosynthesis for primary consumers to utilize, directly or indirectly regulating soil fertility, nutrition cycling and transformation, community productivity, and so on (Cosme 2023), maintaining a beneficial cycle within the ecosystem. The ecological niche differentiation among plant species can reduce the intensity of interspecific competition. In communities with stronger competition, species tend to exhibit a preference for different niches to reduce overlap and interspecific competition, leading to an increase in biodiversity (Zuppinger‐Dingley et al. 2014). Many studies have revealed that the competition for nutrients played a crucial role in determining the coexistence of species (Brose 2008; Katano et al. 2015) and primary consumers may be more inclined to select more energetically efficient plant species (Schneider et al. 2016). The above indicates that we cannot ignore the important role of plant ecological niche indicators in multitrophic level studies.

In addition to considering biotic factors, the impact of abiotic factors on ecosystem functioning should not be overlooked. Elevation influences the developmental diversity and species richness of plant systems through environmental filtering (Galván‐Cisneros et al. 2023), regulating changes in various ecosystem functions (Fu et al. 2020). The diversity of microbial communities, the complexity of co‐occurrence networks, and the multifunctionality of ecosystems all significantly decrease with increasing elevation (Chen et al. 2022). Soil pH, as a dominant factor in explaining multifunctionality and functional group changes, inhibits ecosystem functions and multifunctionality due to soil acidification (Wei et al. 2022), and it also significantly impacts soil biodiversity, thereby affecting ecosystem stability (Chen et al. 2021).

**FIGURE 1:**
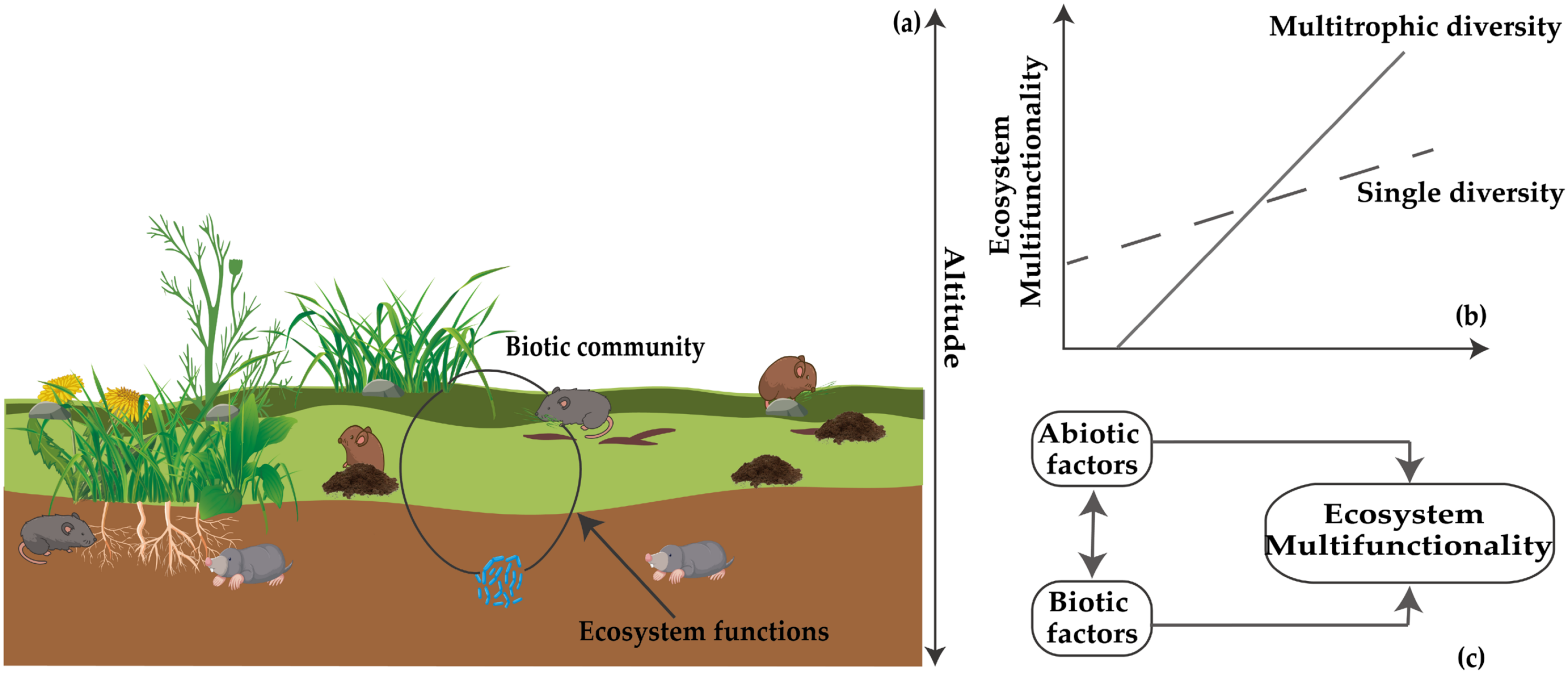
Conceptual framework: (a) the biotic community is composed of three trophic levels—plants, rodents, and microbes. (b) Multitrophic diversity is predicted to enhance ecosystem multifunctionality. (c) Biotic and abiotic variables have direct and indirect effects on ecosystem multifunctionality.

The interactions among biotic factors across multiple trophic levels, as well as abiotic factors, have been shown to influence ecosystem functioning. However, alpine grasslands as the most important ecosystem and natural resource on the Qinghai‐Tibet Plateau, covering over 60% of the region (Zuo et al. 2022), it remains unclear how the diversity of trophic levels and abiotic factors impact the ecosystem multifunctionality of alpine grasslands. To fill these critical knowledge gaps, our study aimed to reveal how multitrophic diversity (including plant diversity, microbial diversity, and rodent diversity) and abiotic variables (including altitude and pH) and their interactions influence ecosystem multifunctionality by investigating the vegetation and soil characteristics of different grassland types in the alpine grassland of Qinghai‐Tibet Plateau. We propose the following hypotheses: (1) the multitrophic diversity of the biotic community will enhance ecosystem multifunctionality more than any single trophic level; (2) the differentiation of plant niche breath is influenced by abiotic factors and ecosystem functioning, thereby affecting the construction of multitrophic level communities; and (3) abiotic factors, particularly altitude and pH, can directly affect ecosystem multifunctionality (Figure 1).

## 2 Materials and Methods

### 2.1 Experimental Site

The experimental site is located in Maqin County, in the southern part of Qinghai Province in the hinterland of the Qinghai–Tibet Plateau, which is situated at the source of the Yellow River and in the core area of the Sanjiangyuan (Table S1). The altitude is 4100 m above sea level, which means the site lies within a typical continental plateau monsoon climate, with strong solar radiation (annual total radiation of 6194 MJ m^−2^), 2493.6 h of sunshine, and no absolute frostless period (Chang et al. 2023). The mean annual temperature is approximately 0.4°C, with low temperatures and large daily temperature differences. The annual precipitation is 531.6 mm, mostly concentrated in June and September. In the last decade, the highest average temperature was 9.9°C and the lowest was −6.1°C, with rain and heat occurring at the same time, which is favorable for pasture growth (Xiang 2023). The total area of Maqin County is 134.60 × 10^4^ hm^2^, of which the grassland area is 117.57 × 10^4^ hm^2^. The usable grassland area is 108.53 × 10^4^ hm^2^, which accounts for 92.3% of the total grassland area, and the grassland type is mainly alpine meadow grass, within which the main dominant plants are *Carex alatauensis*, *C. parvula*, *C. capillifolia*, *C. tibetikobresia*, and *C. hughii* (Dou, Su, and Dekui 2023).

### 2.2 Experimental Design

In 2023, our experiment was conducted in five distinct vegetation types on an alpine grassland (Table S1). Within each vegetation type, we selected five spatial replicates of sampling plots (15 m × 15 m), each separated by a distance of at least 2 km. In each plot, we randomly selected five quadrats (0.5 m × 0.5 m) for the vegetation survey. The aboveground biomass was collected by pruning all plants within the quadrats and putting them in envelopes. Three points were evenly selected on the diagonal of each treatment plot as soil sampling points, and 0–10 cm layers of soil were obtained from the quadrat with a soil drill (diameter: 5 cm). The samples from the same layer were mixed with the three sampling points of each treatment, and then the soil roots were sieved through a 0.5 mm mesh, before packing the soil into soil sample bags and taking them to the laboratory for calculation of the soil nutrient indexes. In addition, soil samples of 0–5 cm were obtained from the quadrats with a soil drill (diameter: 1 cm) near the soil sampling points, mixed evenly, packed into Ziplock bags, labeled, and stored at −80°C in a freezer for microbial sequencing analysis.

In the rodent survey, circular quadrats with a radius of 25 m and similar site conditions were established in different types of grassland over rodent holes. The holes were then plugged and landfilled, and the total number of holes and effective rodent holes were investigated. To reduce sampling error due to the large size of the quadrats, each circular quadrat was divided into three equal sectors along the radius (Figure S1). Rodents were captured using the rope noose method, identified to species, and then released (Appendix S1).

### 2.3 Biotic Variables

The plant diversity indices selected in this study included Margalef (*PS*), Shannon–Wiener (*PH*′), Simpson (*PD*), and Niche breadth (*B*), to estimate the diversity of community structure; the calculation formulae of which are as follows (Wen et al. 2020): rodent diversity indices selected in this study included the number of rodent species (*RS*), number of rodent holes (*NRH*), number of effective holes (*NEH*), and proportion of effective holes (*PEH*). The microbial diversity indices selected in this study included the Chao1 index of bacteria and fungi (*BC* and *FC*), the Shannon–Wiener index of bacteria and fungi (*BH*′ and *FH*′), and the Simpson index of bacteria and fungi (*BD* and *FD*, Appendix S1). Lastly, the multitrophic diversity and species diversity (e.g., plant diversity) were calculated by averaging the standardized diversity indices measured across trophic levels (Wagg et al. 2014; Soliveres et al. 2016; Luo et al. 2022).

PS=S

$${\mathrm{PH}}^{\prime }=-\sum_{i=1}^{S}P_{i}\ln P_{i}$$

$$\mathrm{PD}=1-\sum_{i=1}^{S}P_{i}^{2}$$

$$B_{i}=-\sum_{j}^{r}N_{\mathrm{ij}}\log N_{\mathrm{ij}}$$

where *S* is the number of species; *P*_*i*_ is the relative importance value of species *i*, calculated by the formula (relative abundance + relative height + relative coverage)/3; and *N*_*ij*_ is the frequency of resource utilization of species *i* in sample *j*, normalized by the total frequency of resource utilization of species *i* across all samples: *N*_*ij*_ = *m*_*ij*_/*M*_*i*_, in which *M*_*i*_ = $\sum_{j}^{r}m_{\mathrm{ij}}$, where *m*_*ij*_ is the dominance degree of species *i* on resource *j*, which is equivalent to the importance value of species *i* in the sample, and *r* represents the total number of samples. The larger the value of *B*_*i*_, the wider the niche, and the greater the total amount of resources utilized by the species, indicating a stronger competitive ability.

### 2.4 Abiotic Variables

Abiotic variables included altitude and soil pH, which may affect ecosystem functions or multifunctionality directly or indirectly (Peters et al. 2019).

### 2.5 Ecosystem Functions

Fifteen ecosystem functions were measured. There were moisture content (%, MC), total nitrogen (g kg^−1^, TN), organic carbon (g kg^−1^, OC), total phosphorus (g kg^−1^, TP), alkali hydrolyzed nitrogen (mg kg^−1^, AHN), available phosphorous (mg kg^−1^, AP), ammonium nitrogen (mg kg^−1^, AMN), nitrate nitrogen (mg kg^−1^, NN), the ratio of organic carbon to total nitrogen (C:N), the ratio of organic carbon to total phosphorus (C:P), the ratio of total nitrogen to total phosphorus (N:P), the ratio of organic carbon to total nitrogen to total phosphorus (C:N:P), plant aboveground biomass (g m^−2^, AGB), plant belowground biomass (g m^−2^, BGB), and the ratio of plant aboveground biomass to belowground biomass (A:B). Soil moisture content is a critical factor that can determine biological activity across multiple trophic levels; therefore, we consider it an ecosystem function (Dietrich et al. 2024). Details of the data collection for these different ecosystem functions are provided in Appendix S1.

### 2.6 Ecosystem Multifunctionality

As mentioned above, a total of 15 important ecosystem functions were selected to characterize the ecosystem multifunctionality. Specifically, these functions are good indicators of water conservation (e.g., moisture content), soil fertility (e.g., total nitrogen), nutrition cycling and transformation (e.g., the ratio of organic carbon to total nitrogen), and community productivity (e.g., plant aboveground biomass). Since there is no standardized method for studying ecosystem multifunctionality, we used an intuitive averaging method for evaluation (Hooper and Vitousek 1998; Maestre et al. 2012; Byrnes et al. 2014). We first normalized all ecosystem functions to a comparable range of 0–1, and then the ecosystem functions (EF) were min–max‐transformed: EF = [rawEF − min (rawEF)]/[max(rawEF) − min(rawEF)] (Argens et al. 2023). The EMF and ecosystem functions (e.g., water conservation) were then calculated by averaging the normalized ecosystem functions.

### 2.7 Statistical Analysis

In this study, the effects of different grassland types on biotic (e.g., plant, rodent, and microbial diversity indices) and abiotic variables (e.g., ecosystem functions) were assessed using linear mixed‐effects models with the “lme4” package (Bates et al. 2015). Post hoc tests were conducted employing the “Tukey” method in the “emmeans” package (Dusza et al. 2022). Pearson's correlation analysis was performed on the relationships of species diversity, keystone species, with ecosystem functions. Random Forest modeling, a machine‐learning algorithm, was applied to select the most important species in different grassland types and the most important variables affecting ecosystem multifunctionality with the “rfPermute” package (Archer 2022). The importance of genera abundance was estimated by calculating the percentage increases in the mean squared error (MSE) of variables. Higher values of MSE indicate more significant variables. We also used variation partitioning analysis to quantify the relative importance of three groups of factors as predictors of ecosystem functions and the multifunctionality via the “vegan” package in R (Oksanen et al. 2020). Additionally, we compared the effects of species diversity and multitrophic diversity on ecosystem functions and multifunctionality and evaluated the explanatory power (*R* ^2^) of single and multiple trophic levels from general linear models. All predictors and response variables were standardized to range from 0 to 1 before analysis, including (a) multitrophic diversity, (b) pH, and (c) altitude. Finally, “vegan” and “piecewise SEM” in the R package were used to quantify the effects of biotic and abiotic variables on ecosystem multifunctionality (Lefcheck 2016). To further improve the fit of the models, we created alternative models by progressively removing nonsignificant paths.

## 3 Results

### 3.1 Species Diversity and Composition

**FIGURE 2:**
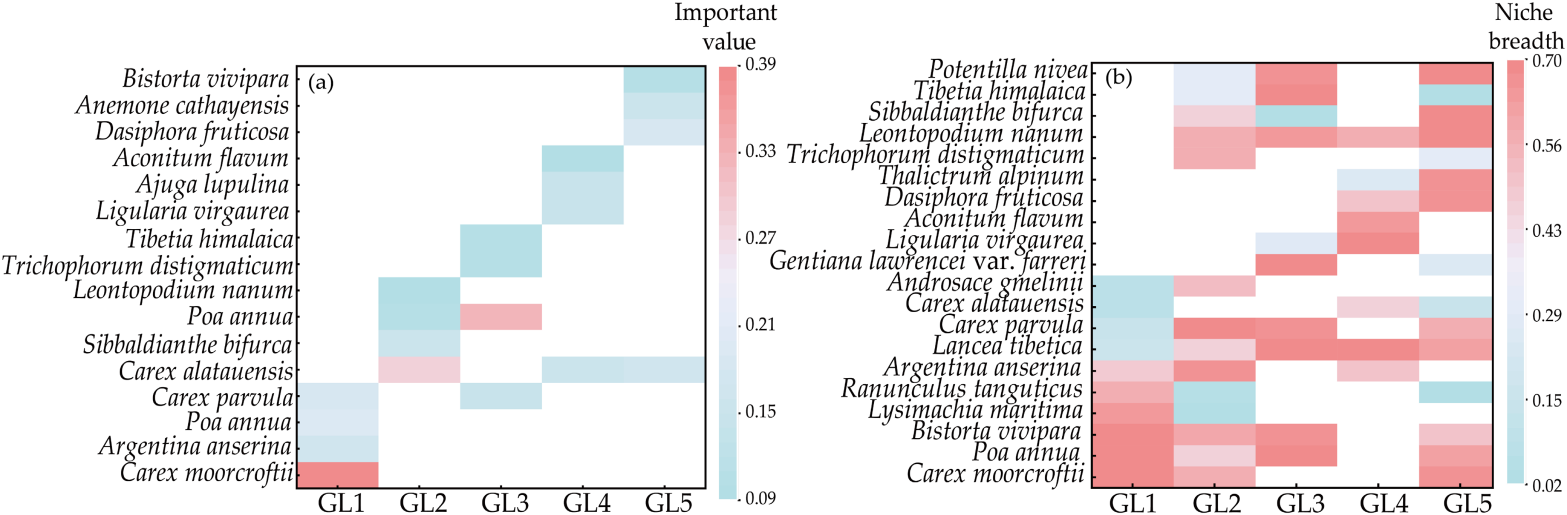
Heat map of the importance value (a) and niche breadth (b) of dominant plants in different grassland types.

Results showed that species diversity indices had significant differences among grassland types. Specifically, the plant diversity indices of GL5 were the highest, and the fungal diversity indices of GL4 were the highest (Figures S2 and S3). The rodent species of GL5 were the highest, with *Ochotona curzoniae* being the absolute dominant group of grassland rodents in the study area (Table S3). Also, there were contrasting linkages among species diversity indices. The number of rodent species was positively correlated with the number of plant species and bacterial Chao1, whereas fungal diversity indices were negatively correlated with plant and rodent diversity indices (Figure S4). Dominant species were composed of different biotic communities among grassland types. Grassland types were classified by the dominant plants as *C. moorcroftii*, *C. alatauensis*, *Poa annua*, *Ligularia virgaurea*, and *C. alatauensis* with *Dasiphora fruticosa* (Figure 2a). The keystone species in the plant community had different niche breadths among sample sites (Figure 2b). The dominant species of microbial and rodent communities changed with the plant community (Figure S5 and Table S3).

### 3.2 Relationship Between Species Diversity and Ecosystem Functions

**FIGURE 3:**
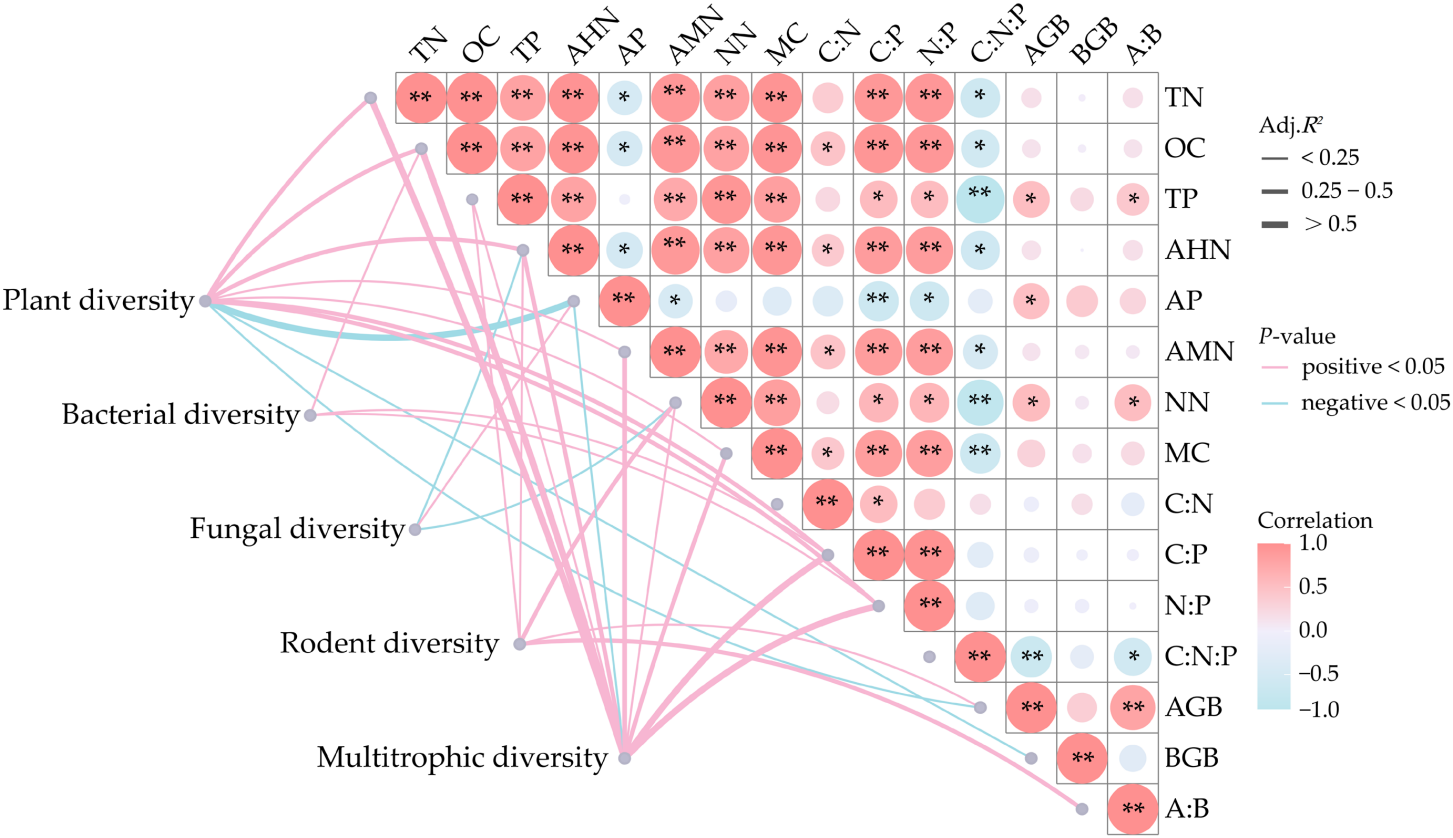
A visualization of a Pearson's correlation matrix of ecosystem functions, and the effects of different trophic diversity on 15 ecosystem functions through linear models. Line thickness is plotted and the value is labeled as Adjusted *R* ^2^ from the linear model. The pink lines indicate positive effects and the blue lines indicate negative effects. A single asterisk (*) and a double asterisk (**) indicate significant relationships via the Pearson's test at *p* < 0.05 and *p* < 0.001, respectively. A:B, ratio of plant aboveground biomass to belowground biomass; AGB, plant aboveground biomass (g m^−2^); AHN, alkali hydrolyzed nitrogen (mg kg^−1^); AMN, ammonium nitrogen (mg kg^−1^); AP, available phosphorous (mg kg^−1^); BGB, plant belowground biomass (g m^−2^); C:N, ratio of organic carbon to total nitrogen; C:N:P, ratio of organic carbon to total nitrogen to total phosphorus; C:P, ratio of organic carbon to total phosphorus; MC, moisture content (%); N:P, ratio of total nitrogen to total phosphorus; NN, nitrate nitrogen (mg kg^−1^); OC, organic carbon (g kg^−1^); TN, total nitrogen (g kg^−1^); TP, total phosphorus (g kg^−1^).

Most keystone species were significantly correlated with ecosystem functions except for the belowground biomass and ratio of organic carbon to total nitrogen (Figure S8). Species diversity indices were significantly correlated with ecosystem functions. Specifically, total nitrogen, organic carbon, alkali hydrolyzed nitrogen, and N: P were positively correlated with plant diversity indices, bacterial Chao1, and the number of rodent species, while available phosphorous was negatively correlated with plant diversity indices, bacterial Chao1, and the number of rodent species (Figure S9). For different trophic level, the plant diversity, bacterial diversity, fungal diversity, and rodent diversity yielded significantly positive and negative relationships with ecosystem functions. When the multitrophic diversity were examined, the most ecosystem functions increased along with increasing multitrophic diversity except for available phosphorus (Figure 3).

### 3.3 Predictors of Major Ecosystem Functions and Multifunctionality

**FIGURE 4:**
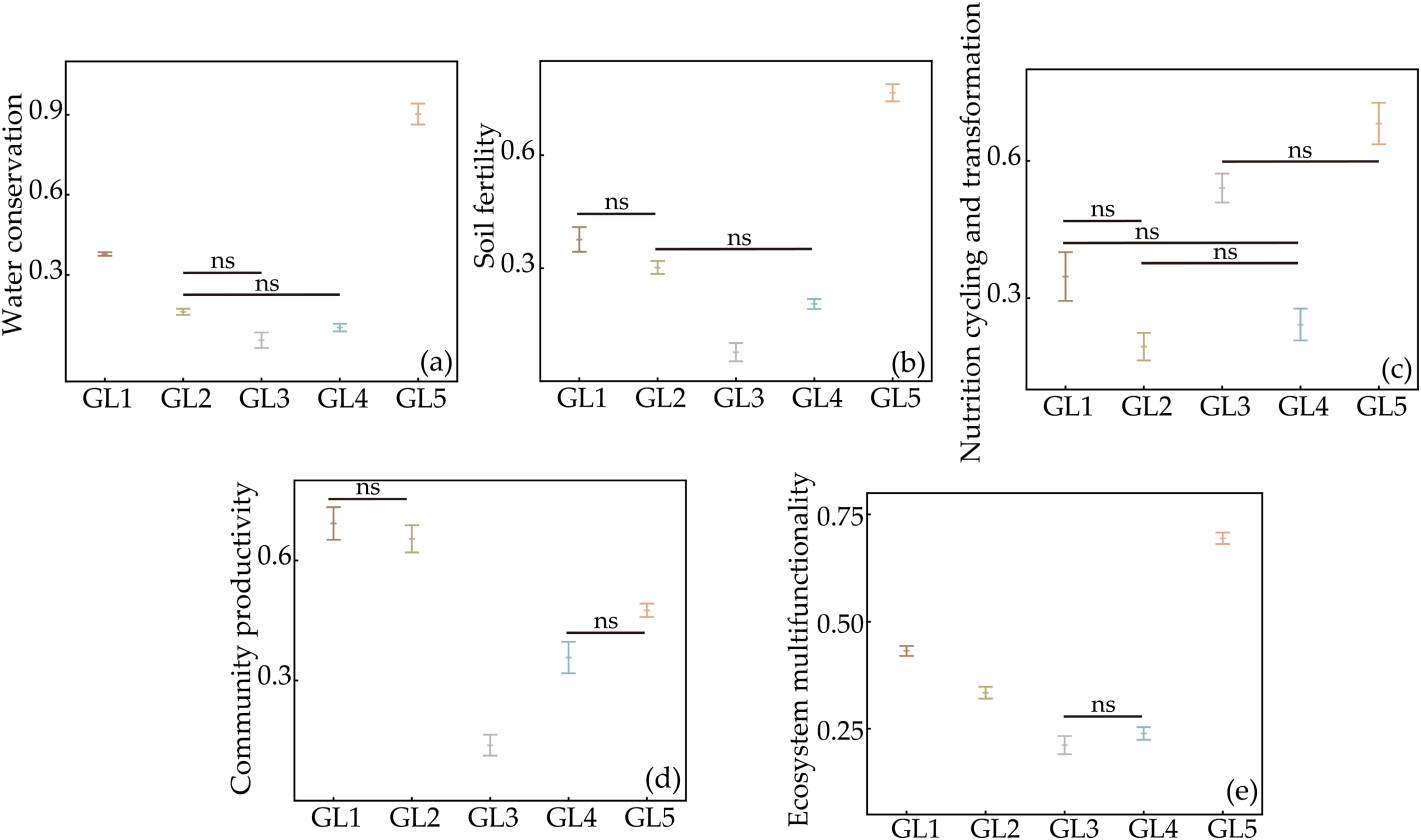
Ecosystem functions in different grassland types: (a) water conservation; (b) soil fertility; (c) nutrition cycling and transformation; (d) community productivity; and (e) ecosystem multifunctionality. “ns” indicates no significant difference, while groups without “ns” notation show significant differences between them (*p* < 0.05).

**FIGURE 5:**
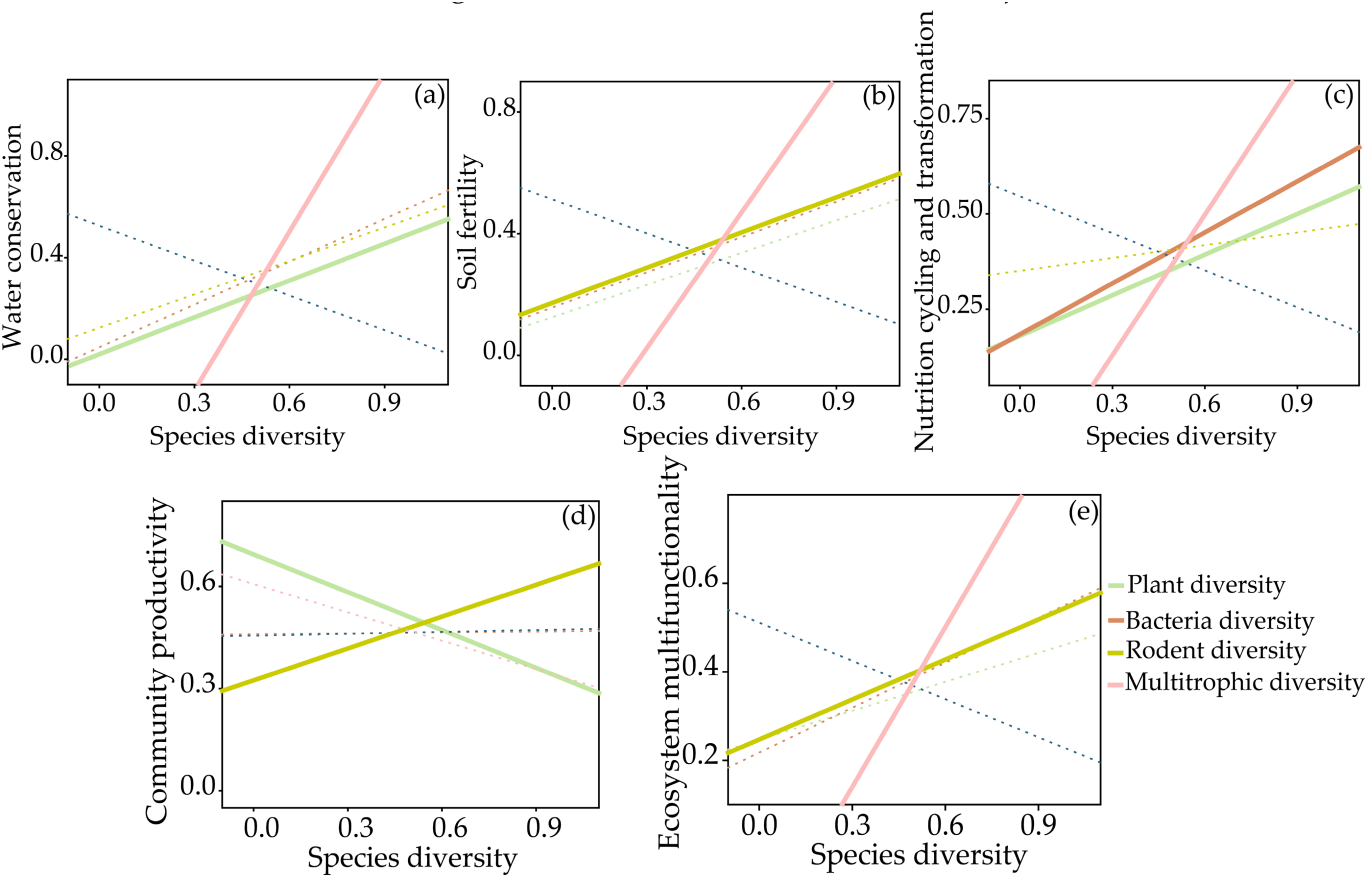
The effects of different trophic species diversity on ecosystem functions: (a) water conservation; (b) soil fertility; (c) nutrition cycling and transformation; (d) community productivity; and (e) ecosystem multifunctionality. The solid lines indicate significant relationship and the dashed lines indicate no significant relationship.

We further grouped these 15 individual ecosystem functions into four major functions and ecosystem multifunctionality (water conservation, soil fertility, nutrition cycling and transformation, and community productivity, Figure 4 and Table S2). A random forest analysis showed that altitude, plant, and rodent diversity indices as predictors could better explain the four major functions and ecosystem multifunctionality (Figure S10). Moreover, the significant predictors of ecosystem functions and multifunctionality were different (Figure 5 and Table S4). A function‐dependent pattern was found among these relationships. For example, plant diversity had positive relationships with water conservation, nutrition cycling and transformation, and community productivity (Figure 5a,c,d), and rodent diversity had positive relationships with soil fertility, community productivity, and ecosystem multifunctionality (Figure 5b,d,e). Among these predictors, multitrophic diversity as a critical one to explain the relationships with functions except for community productivity (Figure 5a–c,e). Notably, it was interesting to observe that multitrophic diversity had a more pronounced positive impact on ecosystem multifunctionality when compared to species diversity within any individual group, which could improve ecosystem multifunctionality (Figure 5e). Overall, ecosystem multifunctionality was better predicted by multitrophic diversity than by other types of diversity.

### 3.4 Contribution of Multitrophic Species Diversity in Explaining Ecosystem Multifunctionality

**FIGURE 6:**
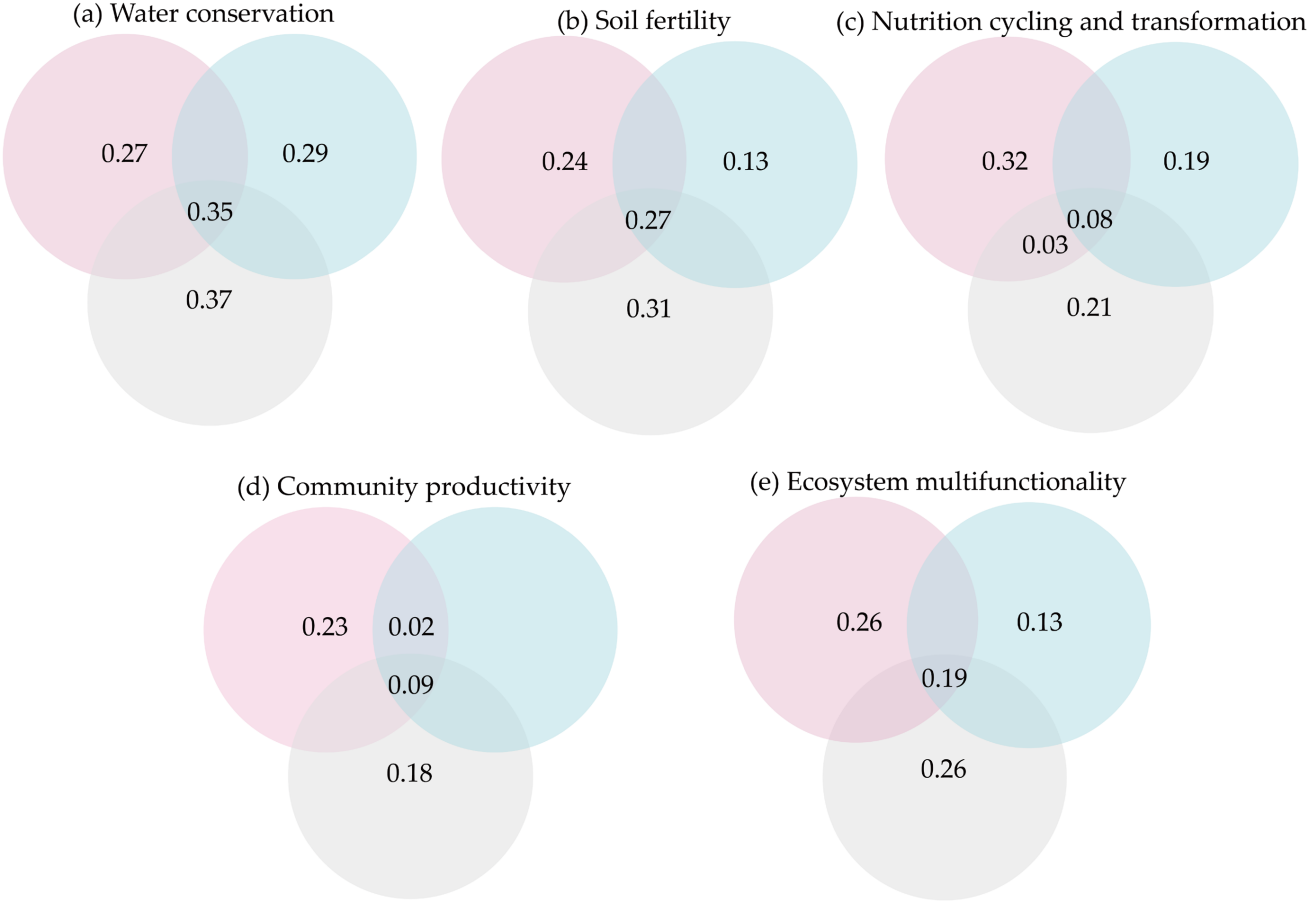
Venn diagrams of the variance of ecosystem functions explained (%) by multitrophic diversity (plant diversity, bacterial diversity, fungal diversity, and rodent diversity), soil environment (pH), and altitude, in which pink circles indicate multitrophic diversity, blue circles indicate soil environment, and gray circles indicate altitude: (a) water conservation; (b) soil fertility; (c) nutrition cycling and transformation; (d) community productivity; and (e) ecosystem multifunctionality.

**FIGURE 7:**
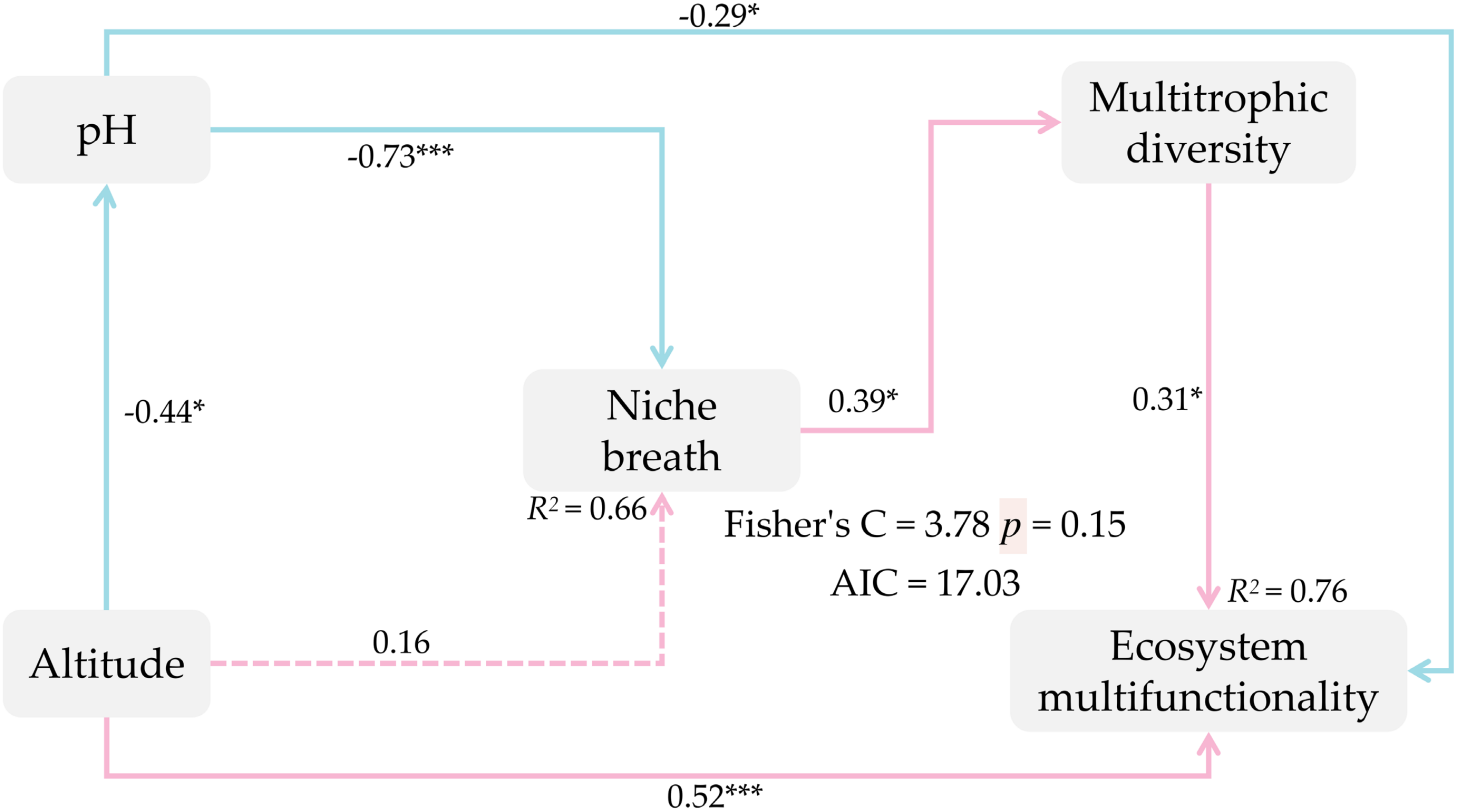
Piecewise SEM describing how biotic and abiotic variables affect ecosystem multifunctionality. Numbers on the arrows represent all the path coefficients and squared multiple correlations (*R* ^2^) in the model. Solid lines indicate significant paths and dashed lines represent paths that were not significant. Positive path coefficients are pink and negative path coefficients are blue. A single asterisk (*) and double asterisk (***) indicate a significant difference between the variables at *p* < 0.05 and *p* < 0.001, respectively.

Variation partitioning analysis was used to quantify the relative importance of biotic variables of multitrophic diversity (plant diversity, bacterial diversity, fungal diversity, and rodent diversity) and the abiotic variables of altitude and soil environment (pH). Multitrophic diversity and abiotic variables (pH and altitude) accounted for 35% and 27% of the variation in water conservation and soil fertility, respectively (Figure 6a,b). Compared with altitude and pH, multitrophic diversity accounted for a higher proportion to predict nutrition cycling and transformation, and community productivity (Figure 6c,d). More importantly, multitrophic diversity and altitude accounted for a similar proportion (26%) of the explained ecosystem multifunctionality (Figure 6e). The results suggested that multitrophic diversity and altitude were more important in supporting specific ecosystem functions. Accordingly, we constructed a Piecewise SEM which explained 76% of variation in ecosystem multifunctionality. Effects of biotic and abiotic variables on ecosystem multifunctionality followed two paths: first, pH and altitude had direct negative and positive effect on ecosystem multifunctionality, respectively; Second, pH had an indirect positive influence on ecosystem multifunctionality via its negative effects on niche breath and multitrophic diversity (Figure 7).

## 4 Discussion

### 4.1 The Diversity and Composition of Different Trophic Levels Link to Ecosystem Functions

Exploring the spatial distribution patterns and mechanisms of biological species diversity on Earth has always been an important part of ecological and biodiversity research. Our study found that different grassland types have significant effects on species diversity loss and soil nutrient enrichment. In grassland types dominated by *Carex alatauensis* with *Dasiphora fruticosa*, species diversity and ecosystem functions were higher (Figures S6 and S7). This is because the increase in a small number of shrub species helps protect herbaceous plants growing under their canopy from grazing by livestock, acting as biological refuges (Oesterheld and Oyarzábal 2004). At the same time, this strengthens the grassland's role in windbreak and sand fixation, which benefits soil improvement (Hu et al. 2023). Moreover, we observed a correlation between species diversity indices of different trophic levels, especially in terms of species richness indices (Figure S4), which is consistent with previous findings (Wang and Xing 2021) indicating that species diversity can be an important indicator reflecting community dynamics. Meanwhile, plant communities undergo changes due to natural disturbances in the form of succession, resulting in changes to the community environment and effects on herbivores, pollinators, and microbes (Beck et al. 2015; Tao et al. 2023). The relationship between plants and microbes can be understood from the mechanism of plant–soil feedback, where different plant communities produce different types of litter and root exudates, affecting soil organic carbon content and thereby changing the composition and structure of soil microbial communities (Smith, Marin‐Spiotta, and Balser 2015). Researchers found that the influence of plant diversity on multifunctionality was indirectly influenced by shifts in soil biodiversity and plant cover (Delgado‐Baquerizo et al. 2020). This underscores the importance of incorporating soil biodiversity into policy and management initiatives aimed at safeguarding the functionality of terrestrial ecosystems on a global scale. The activities of different types and quantities of rodents, through digging and seed dispersal behaviors, have been shown to influence the nutrient characteristics of the soil and plant species (Pech et al. 2007; Wesche, Nadrowski, and Retzer 2007), leading to changes in the composition of the surrounding plant community (Table S3).

Ecosystem functions are closely related to the composition of keystone species in biological communities. The realization of ecosystem functions often depends on certain keystone species in the biological community, such as important predators, pollinators, and major tree species in forests (Raffard et al. 2021; Joshi et al. 2022). Our study found that keystone species in plant, animal, and microbial communities were interconnected with indicators of ecosystem functions (Figure S8). At the same time, our results provided evidence for the theory that ecological niche differentiation is necessary to maintain species coexistence (MacArthur 1970; Turnbull et al. 2016). For example, the similarity of soil functional requirements of some plants (e.g., *Thalictrum aquilegiifolium* var. *sibiricum*, *Sibbaldianthe bifurca*, *Saussurea pulchra*, and *Euphorbia altotibetica*, Figure S8a) and their different ecological niche widths in the community (Figure 1b) suggested that there was obvious competition for resources in the plant community, which affected the differences in the distribution of individual plants in the community. It has been shown that species ecological niche differentiation occurs during the construction of functional groups in grasslands due to environmental factor constraints, resulting in the coexistence of some species and the loss of others (Dray and Legendre 2008), where environmental factors determine which traits are included in functional groups that could be clustered in localized communities (Ter Braak, Cormont, and Dray 2012). Species ecological niche differentiation and interspecific interactions based on functional traits thus play a decisive role in plant community construction and diversity maintenance (Kraft, Valencia, and Ackerly 2008). In different vegetation areas, microbial key species were also subject to limitations on ecosystem functioning (Figures S5 and S8b,c). The *planctomycete_A‐2* belongs to Planctomycetes, which have the ability to utilize diverse carbon compounds and can survive solely on high‐molecular‐weight dextran as their carbon source, thereby participating in the carbon cycle (Boedeker et al. 2017). Consequently, it was unsurprising to find that planctomycete_A‐2 was susceptible to nitrogen and carbon limitation in our study. Meanwhile, *Pseudomonas_frederiksbergensis* has shown potential to solubilize insoluble mineral phosphate (Zeng, Wu, and Wen 2016), which promotes the release of effective phosphorus, and plant uptake of phosphorus plays a significant role in promoting plant growth. We also found that *Pseudomonas_frederiksbergensis* and the total phosphorus content had a highly significant positive correlation. *Xenodidymella_camporesii* and *Verrucaria_viridigrana* belong to the phylum Ascomycota, which has been reported to tolerate stressful conditions such as low nutrient availability, leading to more efficient use of resources in challenging environments (Chen et al. 2017). This could explain the lack of significant correlation with most soil functions.

Rodents are the primary prey of many carnivores, while also consuming plant seeds and fruits, thereby converting plant energy into animal energy and dispersing plant seeds to different locations, promoting biodiversity maintenance (Yu et al. 2023). Our research found that the abundance of root voles was positively correlated with soil total phosphorus content, nitrate nitrogen content, and aboveground biomass (Figure S8d). A previous study suggested that the metabolic activities of root voles produce urea and provide abundant nitrogen resources for the areas surrounding their burrows, thus making the environment suitable for plant survival and growth (Li et al. 2021). Plateau pikas, as rodents mainly active underground, contribute to soil ventilation and water infiltration through gnawing, digging, and excretion behaviors, bringing organic matter and nutrients to the soil surface, enhancing ecosystem functions, and providing favorable conditions for plant growth (Zhang, Zhang, and Liu 2003).

### 4.2 The Pathways of Multitrophic Diversity Drive Ecosystem Multifunctionality

Most studies have begun to focus on changes in ecosystem multifunctionality at different trophic levels. Our study showed that multitrophic‐level diversity had a greater impact on ecosystem multifunctionality than the diversity of a single biome, suggesting that maintaining ecosystem multifunctionality requires the joint participation of biomes (Figures 4, 5, 6), which is consistent with the results of others (Schuldt et al. 2018; Luo et al. 2022; Mori, Isbell, and Cadotte 2023). Therefore, the relationship between biodiversity and ecosystem multifunctionality is more dependent on diversity at the level of multiple communities, and researchers should carry out their analyses at large spatial scales rather than in single communities when considering species diversity.

In addition to this, we found that ecosystem functions such as soil and water conservation, soil fertility, nutrient cycling and transformation, and community productivity were mediated by different single communities in addition to multitrophic level diversity (Figure 4). Due to the similarity in functional traits among different trophic groups and the low redundancy between trophic levels, different trophic groups support different functions (Soliveres et al. 2016; Luo et al. 2022). Plant diversity had a positive effect on water conservation. The increase in water conservation may result from increasing vegetation coverage, which could lead to a reduction in soil evaporation and enhancement of rainfall interception, as well as an increase in soil carbon and nitrogen levels (Figure S9). This is because the presence of soil organic matter can improve soil porosity, thereby boosting both soil water infiltration and retention (Zhu et al. 2019; Zhao et al. 2023). Plant community diversity, on the other hand, had a negative effect on community productivity, which may have been due to the fact that increased plant species richness increased competition among plants, and plant competition has been shown to stimulate an increase in plant litter and root secretions (Laganière et al. 2015). Our study in turn confirmed that there were differences in resource utilization strategies among different keystone species in the plant community, and therefore community productivity can be highly unstable in this situation, producing a downward trend. Soil microbes play an important role in driving EMF (Jing et al. 2015; Delgado‐Baquerizo et al. 2020). The higher the soil bacterial diversity, the more important a role it plays in nutrient cycling and transformation functions, and different microbial species are strongly associated with different ecosystem functions. Maintaining a higher richness of microbial taxa is crucial for supporting increased functional redundancy and diversity, which helps explain why greater biodiversity is necessary to sustain more ecosystem functions (Wagg et al. 2019).

Here, we discovered that considering the characteristics of bacterial communities often proved to be a more effective predictor of ecosystem multifunctionality compared to considering fungal communities (Figure 4). This was due to the distribution of metabolic tasks among microorganisms, creating synergies between microorganisms with distinct physiological characteristics, such as those between fungi and bacteria. This highlights the significance of interconnections between microbial communities in influencing ecosystem functions, suggesting that these hidden synergies may have a broader and more significant ecological impact on soil microbial functions than previously recognized (Kohlmeier et al. 2005; Deveau et al. 2018). Our study also found that soil fertility, grassland productivity, and ecosystem multifunctionality increased significantly with rodent community diversity, and that, in general, mammals at low trophic levels, such as hares and rats, are dependent on food resources and shelter provided by good ecological niches. However, we also suspect that anthropogenic activities may be replacing top‐down ecological effects that are partially independent of top predators. These ecological effects are not mediated by top predators but directly affect small mammals (Smiley et al. 2020; She et al. 2023).

Changes in the diversity of multiple trophic levels can be explained by variations in the aboveground plant niche breadths, and as the niche expands, the diversity of multiple trophic levels and ecosystem multifunctionality also increase (Figure 6). The response of plant species to interspecific competition involves altering their niches to reduce overlap with other species, making them more complementary in resource utilization (Eisenhauer et al. 2019). Plant communities with high diversity select for enhanced niche differentiation among species, reducing interspecific competition without increasing intraspecific competition. This mechanism may strengthen the relationship between biodiversity and ecosystem functioning (Amyntas et al. 2023). The relationship between plant diversity and ecosystem functioning depends not only on interactions between plants but also on interactions within and between different trophic levels of the food chain (Barnes et al. 2020; Albert et al. 2022). This emphasizes the importance of plants as primary producers in connecting aboveground consumers and belowground decomposers, thus influencing changes in multitrophic‐level biological communities.

### 4.3 Altitude and pH Influence Ecosystem Multifunctionality

Biodiversity is not the sole or primary driving factor of ecosystem multifunctionality (Giling et al. 2019); climate and abiotic factors also drive ecosystem functions (Grytnes and McCain 2013). We found that biotic factor of altitude and pH directly explained changes in ecosystem multifunctionality (Figures 5 and 6), which aligned with previous evidence (Hu et al. 2020; Luo et al. 2022). Altitude serves as a predictive factor for patterns of species diversity and community composition under environmental changes. With increasing altitude, temperature decreases, solar radiation increases, and wind strength intensifies, putting significant stress on the physiological and survival strategies of species (Galván‐Cisneros et al. 2023). Studies had shown that ecosystem multifunctionality significantly decreases with increasing altitude (Chen et al. 2022), which is contrary to our results. The reason for this discrepancy may be that the relationship between altitude and ecosystem multifunctionality is not simply linear but follows a single‐peak curve. Research on the dependence of ecosystem multifunctionality on altitude in the Qinghai‐Tibet Plateau indicated a positive correlation between altitude and ecosystem multifunctionality at altitudes below approximately 3900 m (Wang, Sun, and Lee 2023). Our study sites were located near 3900 m, which might explain why there was a positive correlation between altitude and ecosystem multifunctionality in our findings. Soil pH directly affects processes such as mineral weathering, organic matter mineralization, and humification in soils, which have a significant impact on the status of nutrient ions in soils. Most studies have confirmed that soil acidification reduces ecosystem functionality (Delgado‐Baquerizo et al. 2016; Wei et al. 2022), which is inconsistent with our research findings. Through our study of sample points, we found that high‐altitude grasslands with lower pH and higher ecosystem multifunctionality show obvious shrub encroachment. The pH content of shrub‐encroached high‐altitude grasslands tends to be acidic (Ma et al. 2022), and shrub encroachment can enhance ecosystem functionality, specifically in terms of carbon sequestration, soil fertility, and hydrological functions (Ding and Eldridge 2023). These results support our conclusion.

## 5 Conclusions

In summary, our results indicate that key species at different trophic levels are interconnected with ecosystem functions, leading to species niche differentiation within communities and influencing biodiversity patterns. The interconnections of diversity at a single trophic level, particularly richness indices, highlight the importance of biodiversity in maintaining food web structures and functions within biological communities. The greater the number of species in an ecosystem, the more diverse the food base supply, ensuring ecosystem stability. Furthermore, we confirmed the role of multitrophic diversity, namely biodiversity (i.e., plant diversity, bacterial diversity, and rodent diversity), in maintaining ecosystem functions, further emphasizing the critical importance of conserving biodiversity in sustaining ecosystem functions. Finally, abiotic factors such as altitude alter ecosystem multifunctionality by affecting soil conditions, and multitrophic diversity directly impacts ecosystem multifunctionality. Therefore, in the face of increasing human activities and climate change, the maintenance of multifunctionality through multitrophic diversity is crucial and requires our utmost attention.

## Author Contributions

**Hongye Su:** conceptualization (lead), data curation (lead), formal analysis (lead), investigation (equal), methodology (equal), project administration (equal), resources (equal), software (lead), validation (lead), visualization (lead), writing – original draft (lead), writing – review and editing (equal). **Zhen Wang:** conceptualization (equal), writing – review and editing (supporting). **Li Ma:** data curation (equal), visualization (equal). **Ruimin Qin:** investigation (equal). **Tao Chang:** investigation (equal). **Zhonghua Zhang:** data curation (equal), visualization (equal). **Junfei Yao:** investigation (equal). **Xudong Li:** investigation (equal). **Shan Li:** investigation (equal). **Xue Hu:** methodology (supporting), visualization (supporting). **Jingjing Wei:** investigation (supporting). **Fang Yuan:** methodology (supporting), visualization (supporting). **Haze Adi:** investigation (supporting). **Zhengchen Shi:** investigation (supporting). **Honglin Li:** conceptualization (supporting), writing – review and editing (supporting). **Huakun Zhou:** funding acquisition (lead), project administration (lead), resources (lead), supervision (lead).

## Conflicts of Interest

The authors declare no conflicts of interest.

## Supporting information


Appendix S1

## Data Availability

Data will be made available on request.
