# Supplementary material for: Multitrophic Diversity of the Biotic Community Drives Ecosystem Multifunctionality in Alpine Grasslands
Source: Ecol Evol. 2024 Nov 5;14(11):e70511. doi: 10.1002/ece3.70511 (PMC11538076; doi:10.1002/ece3.70511)
Supplement: Supplementary file 1 — Appendix S1 [file ECE3-14-e70511-s001.docx]

**Appendix S1**

**Materials and methods**

**Plant** We conducted a strata inventory of the plant communities within each quadrat, and recorded the name, coverage, abundance, and height of plant species. Each index was investigated as follows: coverage was obtained by visual estimation to obtain total community coverage and species coverage; abundance was obtained by investigating the number of plants; natural height was measured by randomly selecting five plants of the same species (if there were less than 5 plants, all of them were measured).

**Rodent** Due to the high vigilance of rodents, they exhibit multiple alert behaviors during the predation process, making it difficult to capture them. Therefore, a hole-plugging and hole-opening method was used for population estimation: all burrow openings within the monitoring plot were blocked (holes of plateau zokor needed to be opened) and **the number of rodent holes (NRH)** were recorded. After 3 consecutive days at 12:00~14:00 every day to investigate the new holes thrown open by rodents and then re-fill them, the new holes thrown open by rodents are the effective holes for that day, and **the number of effective holes** for each sample site are the average of the 3 consecutive days. **The number of effective holes** **(NEH)** in each sample site was determined as the average of three consecutive days. The ratio of the effective number of holes to the number of holes is **the proportion of effective holes (PEH).** Rodents (e.g., root vole, plateau pika, and gansu pika) were captured using string nooses method (Qu et al., 2019) which were anchored into the soil with iron wire (after observing the basic characteristics, we released rodents), and **the number of rodent species (RS)** were counted by arranging the traps for three consecutive days in effective holes.

**Microbes** Microbial diversity composition profiling sequencing targeted microbial characteristic sequences of bacterial 16S rRNA gene variable regions and fungal ITS internal transcribed spacers, and reflected the composition information and abundance of various microbial species in the microbial community through detection of sequence variation and quantity. Total DNA was extracted using the EZNA soil DNA kit (Omega). Initial PCR amplifications used 338F/806R and ITS5F/ITS2 primer pairs to target the V3-V4 region of 16S rRNA and ITS1, respectively. The PCR conditions were as follows: 2 min initial denaturation at 98 °C; 25 cycles at 98 °C for 15 s, 55 °C for 30 s and 72 °C for 30 s; and a 30-s final extension at 72 °C. The amplicons were purified using AxyPrep DNA GelExtraction Kit (AxyPrep) and then sequenced on the Illumina NovaSep (PE-250) in Personalbio Company (Shanghai) (Wu et al., 2019). Microbiome bioinformatics were performed with QIIME 2 2019.4 (Bolyen et al., 2019) and then chimera removed using the DADA2 plugin (Callahan et al., 2016). The final output, which was a table of amplicon sequence variants (ASVs) and a random rarefaction of ASVs table was calculated the abundance of bacteria and fungi.

**Chao1 (BC and FC**) First proposed by Chao, the estimation of species richness in a community is calculated by counting the number of ASVs (Amplified Sequence Variants/Operational Taxonomic Units) that are detected only once or twice, referred to as "Singletons" and "Doubletons," respectively. This provides an estimate of the actual number of species present in the community (Chao, 1984).

**Shannon-Wiener diversity index (BH’ and FH’**) By taking into account both the richness and evenness of a community (Shannon, 1948) .

**Simpson index (BD and FD**) A commonly used diversity index to evaluate community diversity by calculating the probability that two randomly sampled individuals (sequences) from the community belong to different species (ASVs) (Simpson, 1949).

**Moisture content (MC)** was determined by measuring the fresh weight (FW) of the sample, then drying at 105 °C, and measuring the dry weight (DW), the calculation formula is as follows:

MC= (FW - DW) / DW

**Total nitrogen** **(TN)** was determined by Kjeldahl acid-digestion method.

**Organic carbon (OC)** concentrations were evaluated using H_2_SO_4_–K_2_Cr_2_O_7_ oxidation.

**Total phosphorus (TP)** was determined by colorimetric method.

**Alkali hydrolyzed nitrogen** **(AHN)** was determined by alkaline nitrogen diffusion method.

**Available phosphorus (AP)** was extracted by 0.5 mol/L NaHCO_3_.

**Ammonium nitrogen (AMN)** was determined by ultraviolet spectrophotometry.

**Nitrate nitrogen (NN)** was determined by Nasser’s reagent spectrophotometry.

**pH** was measured using a pH meter (FE28K, Mettler Toledo, Shanghai) in the soil suspension of 1:5 soil/water (distilled water) (Wang et al., 2014a; Wang et al., 2014b).

**Aboveground biomass (AGB)** Determination of aboveground biomass required drying the plants at 65°C for 48h to constant weight and weighing.

**Belowground biomass (BGB)** The soil was sieved to separate the roots from the soil, the roots were cleaned and then dried at 65°C for 48h to a constant weight, weighed, and then the belowground biomass per unit area was calculated by comparing the root weights of the two layers to the area of the three augers.

**Reference**

Bolyen, E., Rideout, J.R., Dillon, M.R., Bokulich, N.A., Abnet, C.C., Al-Ghalith, G.A., Alexander, H., Alm, E.J., Arumugam, M., Asnicar, F., Bai, Y., Bisanz, J.E., Bittinger, K., Brejnrod, A., Brislawn, C.J., Brown, C.T., Callahan, B.J., Caraballo-Rodriguez, A.M., Chase, J., Cope, E.K., Da Silva, R., Diener, C., Dorrestein, P.C., Douglas, G.M., Durall, D.M., Duvallet, C., Edwardson, C.F., Ernst, M., Estaki, M., Fouquier, J., Gauglitz, J.M., Gibbons, S.M., Gibson, D.L., Gonzalez, A., Gorlick, K., Guo, J., Hillmann, B., Holmes, S., Holste, H., Huttenhower, C., Huttley, G.A., Janssen, S., Jarmusch, A.K., Jiang, L., Kaehler, B.D., Kang, K.B., Keefe, C.R., Keim, P., Kelley, S.T., Knights, D., Koester, I., Kosciolek, T., Kreps, J., Langille, M.G.I., Lee, J., Ley, R., Liu, Y.X., Loftfield, E., Lozupone, C., Maher, M., Marotz, C., Martin, B.D., McDonald, D., McIver, L.J., Melnik, A.V., Metcalf, J.L., Morgan, S.C., Morton, J.T., Naimey, A.T., Navas-Molina, J.A., Nothias, L.F., Orchanian, S.B., Pearson, T., Peoples, S.L., Petras, D., Preuss, M.L., Pruesse, E., Rasmussen, L.B., Rivers, A., Robeson, M.S., 2nd, Rosenthal, P., Segata, N., Shaffer, M., Shiffer, A., Sinha, R., Song, S.J., Spear, J.R., Swafford, A.D., Thompson, L.R., Torres, P.J., Trinh, P., Tripathi, A., Turnbaugh, P.J., Ul-Hasan, S., van der Hooft, J.J.J., Vargas, F., Vazquez-Baeza, Y., Vogtmann, E., von Hippel, M., Walters, W., Wan, Y., Wang, M., Warren, J., Weber, K.C., Williamson, C.H.D., Willis, A.D., Xu, Z.Z., Zaneveld, J.R., Zhang, Y., Zhu, Q., Knight, R., Caporaso, J.G., 2019. Reproducible, interactive, scalable and extensible microbiome data science using QIIME 2. Nat Biotechnol 37, 852-857.

Callahan, B.J., McMurdie, P.J., Rosen, M.J., Han, A.W., Johnson, A.J.A., Holmes, S.P., 2016. DADA2: High-resolution sample inference from Illumina amplicon data. Nature Methods 13, 581-583.

Chao, A., 1984. Non-parametric estimation of the classes in a population. Scandinavian Journal of Statistics 11, 265-270.

Qu, J., Reale, D., Fletcher, Q.E., Zhang, Y., 2019. Among-population divergence in personality is linked to altitude in plateau pikas *(Ochotona curzoniae)*. Front Zool 16, 26.

Shannon, C.E., 1948. A mathematical theory of communication. The Bell System Technical Journal 27, 379-423.

Simpson, E.H., 1949. Measurement of Diversity. Nature 163, 688-688.

Wang, L., Sun, X., Li, S., Zhang, T., Zhang, W., Zhai, P., 2014a. Application of organic amendments to a coastal saline soil in north China: effects on soil physical and chemical properties and tree growth. PLoS One 9, e89185.

Wang, X., Dong, S., Yang, B., Li, Y., Su, X., 2014b. The effects of grassland degradation on plant diversity, primary productivity, and soil fertility in the alpine region of Asia's headwaters. Environ Monit Assess 186, 6903-6917.

Wu, Y., Cai, P., Jing, X., Niu, X., Ji, D., Ashry, N.M., Gao, C., Huang, Q., 2019. Soil biofilm formation enhances microbial community diversity and metabolic activity. Environ Int 132, 105116.

Table S1 Experimental site

| **Experimental site** | **Vegetation type** | **Altitude/m** | **Longitude** | **Latitude** |
| --- | --- | --- | --- | --- |
| GL1 | *Carex moorcroftii* | 3950 | 100°28′57″ | 34°21′15″ |
| GL2 | *Carex alatauensis* | 3800 | 100°16′33″ | 34°24′12″ |
| GL3 | *Poa annua* | 4030 | 100°28′17″ | 34°21′19″ |
| GL4 | *Ligularia virgaurea* | 4110 | 100°28′0″ | 34°20′7″ |
| GL5 | *Carex alatauensis with Dasiphora fruticosa* | 3960 | 100°28′42″ | 34°21′9″ |

Table S2 Effects of different grassland types on biotic variables and ecosystem functions

|  | **SS** | **MS** | **NumDF** | ***F-*value** | ***P*** |
| --- | --- | --- | --- | --- | --- |
| Number of species (Plant) | **126.64** | **31.66** | 4 | 56.536 | **3.079e-09** |
| Shannon-Winener (Plant) | **1.4065** | **0.35164** | 4 | 6.9283 | **0.001956** |
| Simpson (Plant) | **0.075704** | **0.018926** | 4 | 9.5974 | **0.0001684** |
| Chao1 (Bacteria) | 1239868 | 309967 | 4 | 2.1292 | 0.12 |
| Shannon (Bacteria) | 0.1331 | 0.033274 | 4 | 2.5674 | 0.07 |
| Simpson (Bacteria) | **4.264e-07** | **1.066e-07** | 4 | 4.5169 | **0.009207** |
| Chao1 (Fungi) | **12657** | **3164.3** | 4 | 5.4934 | **0.003762** |
| Shannon (Fungi) | **2.1589** | **0.53972** | 4 | 3.9276 | **0.01639** |
| Simpson (Fungi) | 0.003 | 0.000866 | 4 | 2.5322 | 0.07247 |
| Water conservation | **2.4253** | **0.60631** | 4 | 221.71 | **2.934e-16** |
| Soil fertility | **1.3546** | **0.33864** | 4 | 127.62 | **6.165e-14** |
| Nutrition cycling and transformation | **0.84547** | **0.21137** | 4 | 26.459 | **9.681e-08** |
| Community productivity | **1.0278** | **0.25695** | 4 | 49.133 | **8.665e-09** |
| Ecosystem multifunctionality | **0.75925** | **0.18981** | 4 | 158.75 | **7.533e-15** |
| Total nitrogen g·kg^-1^ | **187.91** | **46.977** | 4 | 336.65 | **3.219e-15** |
| Origic carbon g·kg^-1^ | **26565** | **6641.3** | 4 | 244.62 | **3.982e-14** |
| Total phosphorus g·kg^-1^ | **0.69122** | **0.17281** | 4 | 40.722 | **3.393e-08** |
| Alkali hydrolyzed nitrogen mg·kg^-1^ | **495179** | **123795** | 4 | 83.411 | **3.492e-12** |
| Available phosphorous mg·kg^-1^ | **311.23** | **77.808** | 4 | 65.072 | **3.52e-11** |
| Ammonium nitrogen mg·kg^-1^ | **24.756** | **6.1889** | 4 | 103.35 | **4.617e-13** |
| Nitrate nitrogen mg·kg^-1^ | **38111** | **9527.6** | 4 | 70.071 | **1.775e-11** |
| Moisture content % | **13154** | **3288.4** | 4 | 221.63 | **2.945e-16** |
| C:N | **6.7105** | **1.6776** | 4 | 4.3487 | **0.01082** |
| C:P | **14860** | **3715.1** | 4 | 68.977 | **6.975e-10** |
| N:P | **94.282** | **23.571** | 4 | 57.518 | **2.709e-09** |
| C:N:P | **351.6** | **87.901** | 4 | 50.48 | **7.105e-09** |
| A:B | **0.21592** | **0.053979** | 4 | 8.5075 | **0.0003525** |
| pH | **10.836** | **2.7089** | 4 | 107.67 | **3.128e-13** |
| Aboveground biomass g/m^2^ | **451390** | **112847** | 4 | 48.185 | **9.993e-09** |
| Belowground biomass g/m^2^ | 246244 | 61561 | 4 | 1.7172 | 0.1857 |
| Number of rodent holes/pcs·ha^-1^ | **14691939** | **3672985** | 4 | 26.253 | **2.765e-05** |
| Number of effective holes/pcs·ha^-1^ | **797075** | **199269** | 4 | 34.391 | **4.361e-05** |
| Proportion of effective holes/% | **280.06** | **70.015** | 4 | 2236.1 | **3.188e-12** |

Table S3 Composition of small rodent community (mean ± SE)

| **Experimental site** | **Vegetation type** | **Number of rodent holes/pcs·ha^-1^** | **Number of effective holes/pcs·ha^-1^** | **Proportion of effective holes/%** | **Species** |
| --- | --- | --- | --- | --- | --- |
| GL1 | *Carex moorcroftii* | 768±30.62 | 120±5.60 | 15.67±0.12 | Root vole (*Microtus oeconomus*) *and* Plateau pika (*Ochotona curzoniae*) |
| GL2 | *Carex alatauensis* | 3542±398.37 | 754±89.21 | 21.28±0.13 | Plateau pika *and* Gansu pika (*Ochotona cansus*) |
| GL3 | *Poa annua* | 1139±77.82 | 234±14.51 | 20.50±0.14 | Plateau pika |
| GL4 | *Ligularia virgaurea* | 1773±70.61 | 163±5.90 | 9.18±0.03 | Plateau pika |
| GL5 | *Carex alatauensis with Dasiphora fruticosa* | 2477±249.99 | 396±40.55 | 15.99±0.03 | Root vole*,* Plateau pika and Plateau zokor (*Eospalax baileyi*) |

Table S4 The effects of different trophic diversity on ecosystem functions. The character

with bold indicate the significant effect (*P* < 0.05).

| **Ecosystem functions** | **Predictor** | **Slope** | **Adj.R^2^** | **Estimate** | **SE** | ***t-*value** | ***P*** |
| --- | --- | --- | --- | --- | --- | --- | --- |
| Water conservation | Plant diversity | **0.483** | **0.129** | **0.48282** | **0.22627** | **2.134** | **0.0437** |
|  | Bacterial diversity | 0.561 | 0.081 | 0.5612 | 0.3186 | 1.761 | 0.0915 |
|  | Fungal diversity | -0.457 | 0.047 | -0.4570 | 0.3095 | -1.476 | 0.1534 |
|  | Rodent diversity | 0.439 | 0.116 | 0.4389 | 0.2154 | 2.038 | 0.0533 |
|  | Multitrophic diversity | **2.081** | **0.366** | **2.0815** | **0.5404** | **3.852** | **0.000813** |
| Soil fertility | Plant diversity | 0.354 | 0.119 | 0.3536 | 0.1714 | 2.063 | 0.0506 |
|  | Bacterial diversity | 0.387 | 0.060 | 0.3866 | 0.2427 | 1.593 | 0.125 |
|  | Fungal diversity | -0.374 | 0.063 | -0.3740 | 0.2312 | -1.618 | 0.119349 |
|  | Rodent diversity | **0.386** | **0.174** | **0.3859** | **0.1569** | **2.460** | **0.0218** |
|  | Multitrophic diversity | **1.484** | **0.323** | **1.4844** | **0.4206** | **3.529** | **0.0018** |
| Nutrition cycling and transformation | Plant diversity | **0.355** | **0.187** | **0.35535** | **0.13918** | **2.553** | **0.0178** |
|  | Bacterial diversity | **0.446** | **0.150** | **0.4460** | **0.1950** | **2.287** | **0.0318** |
|  | Fungal diversity | -0.324 | 0.068 | -0.3239 | 0.1948 | -1.663 | 0.11 |
|  | Rodent diversity | 0.111 | -0.018 | 0.11148 | 0.14717 | 0.757 | 0.456444 |
|  | Multitrophic diversity | **1.272** | **0.333** | **1.2717** | **0.3528** | **3.605** | **0.00149** |
| Community productivity | Plant diversity | **-0.370** | **0.178** | **-0.3703** | **0.1487** | **-2.490** | **0.0204** |
|  | Bacterial diversity | 0.009 | -0.043 | 0.00884 | 0.22960 | 0.039 | 0.969620 |
|  | Fungal diversity | 0.019 | -0.043 | 0.01854 | 0.21907 | 0.085 | 0.933287 |
|  | Rodent diversity | **0.311** | **0.132** | **0.31128** | **0.14440** | **2.156** | **0.041803** |
|  | Multitrophic diversity | -0.277 | -0.028 | -0.2772 | 0.4654 | -0.596 | 0.5572 |
| Ecosystem multifunctionality | Plant diversity | 0.218 | 0.068 | 0.21790 | 0.13155 | 1.656 | 0.1112 |
|  | Bacterial diversity | 0.339 | 0.099 | 0.33850 | 0.17718 | 1.910 | 0.0686 |
|  | Fungal diversity | -0.288 | 0.070 | -0.28768 | 0.17183 | -1.674 | 0.108 |
|  | Rodent diversity | **0.301** | **0.195** | **0.30131** | **0.11553** | **2.608** | **0.015722** |
|  | Multitrophic diversity | **1.203** | **0.356** | **1.1152** | **0.3125** | **3.568** | **0.00163** |


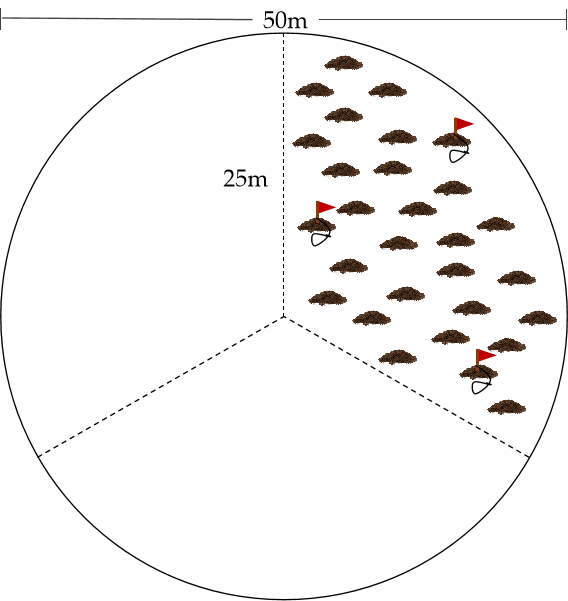


Figure S1 Schematic diagram of survey methods for rodents (Red flag represents effective holes).





Figure S2 Species diversity indices in different grassland types (a. Number of species, b. Shannon-Wiener index, c. Simpson index). *, **, and *** indicate a significant relationship with Pearson test at *P* < 0.05, *P* < 0.01, *P* < 0.001 level respectively.





Figure S3 Microbial diversity indices in different grassland types (a. Bacterial diversity indices, b. Fungal diversity indices). *, **, and *** indicate a significant relationship with Pearson test at *P* < 0.05, *P* < 0.01, *P* < 0.001 level respectively.


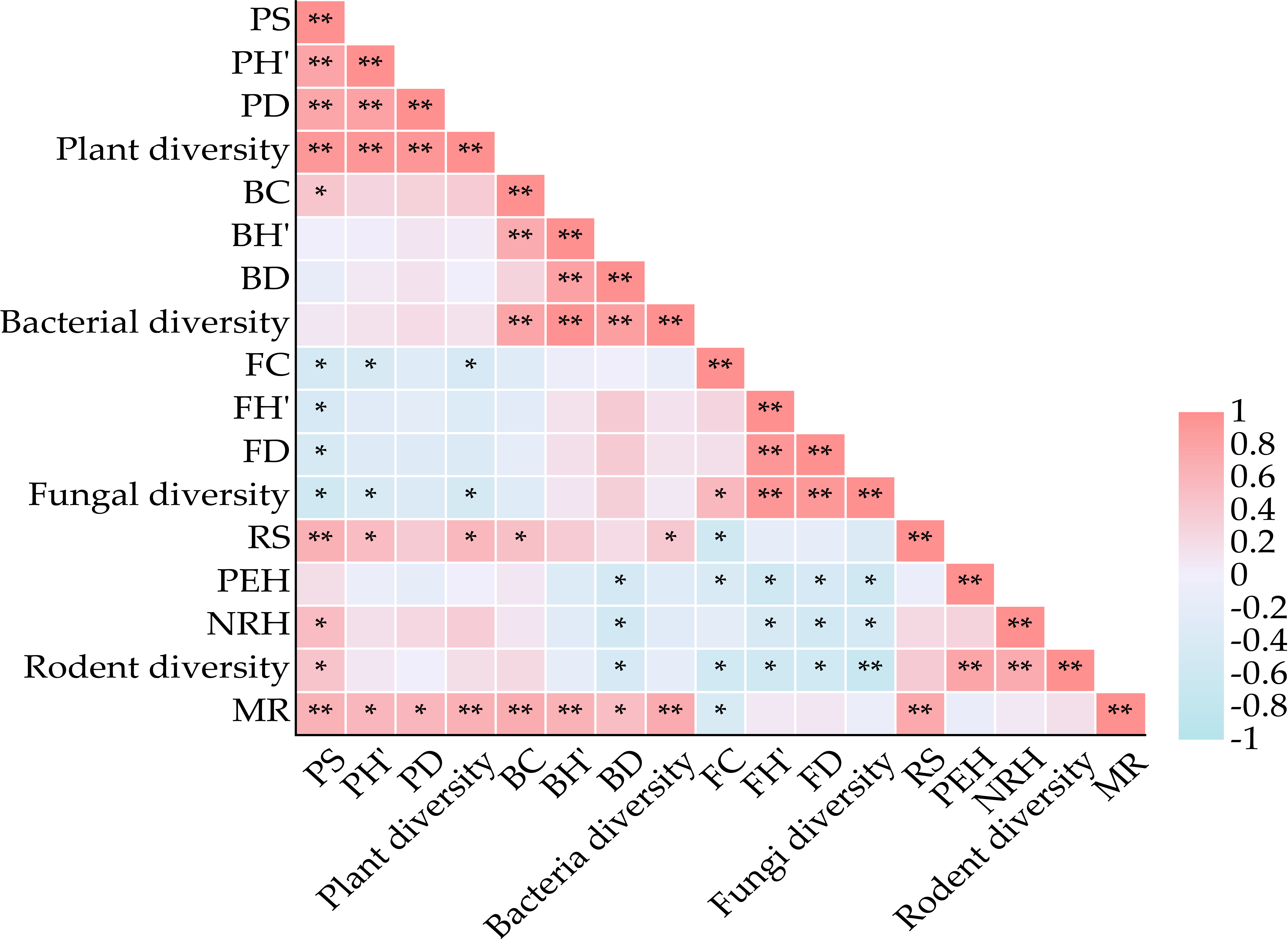


Figure S4 Pearson correlation between species diversity. *, **, indicate a significant relationship with Pearson test at *P* < 0.05, *P* < 0.001 level respectively. ”PS”, “PH”, ”PD”, ”BC”, ”BH’”, ”BD”, ”FC”, ”FH’”, ”FD”, ”RS”, ”PEH”, ”NRH”, and “MR” represent “Plant number of species”, “plant Shannon-Wiener index”, “plant Simpson”, “bacteria Chao1 index”, “bacteria Shannon-Wiener index”, “bacteria Simpson index”, “fungi Chao1 index”, “fungi Shannon-Wiener index”, “fungi Simpson index”, “rodent number of species”, ”Proportion of effective holes %”, “Number of rodent holes pcs·ha^-1^” and “multitrophic diversity” respectively.


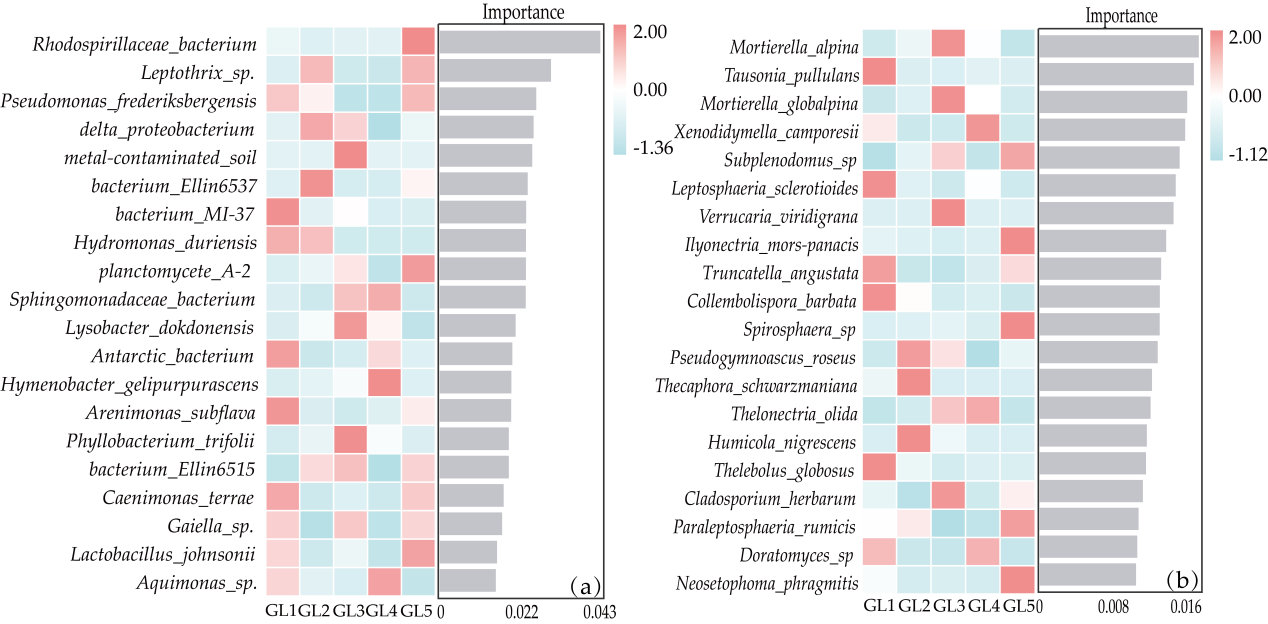


Figure S5 Heat map of the distribution of abundance of dominant microbial species in different grassland types and random forest analysis of the relative importance of dominant microbial species (Top 20 species in terms of importance; a. bacteria, b. fungi).


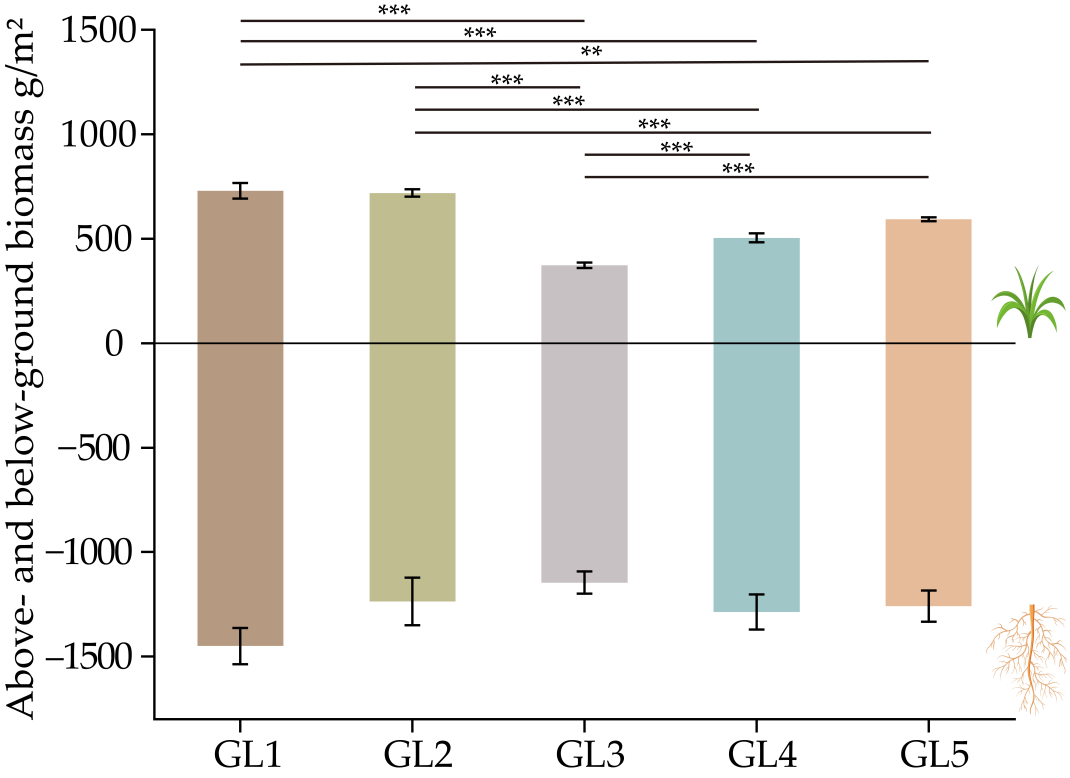


Figure S6 Above- and below- ground biomass in different grassland types. *, **, and *** indicate a significant relationship with Pearson test at *P* < 0.05, *P* < 0.01, *P* < 0.001 level respectively.


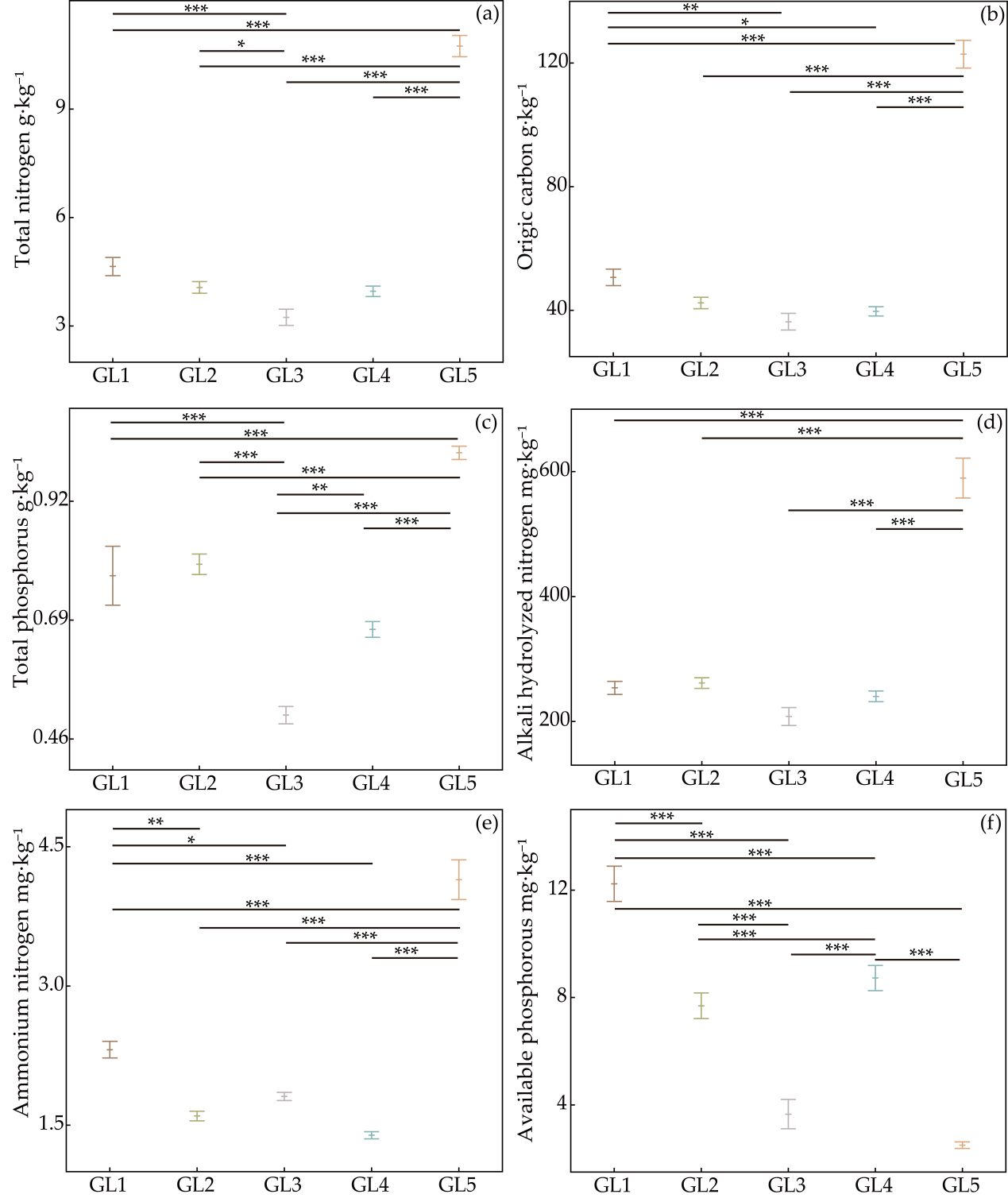


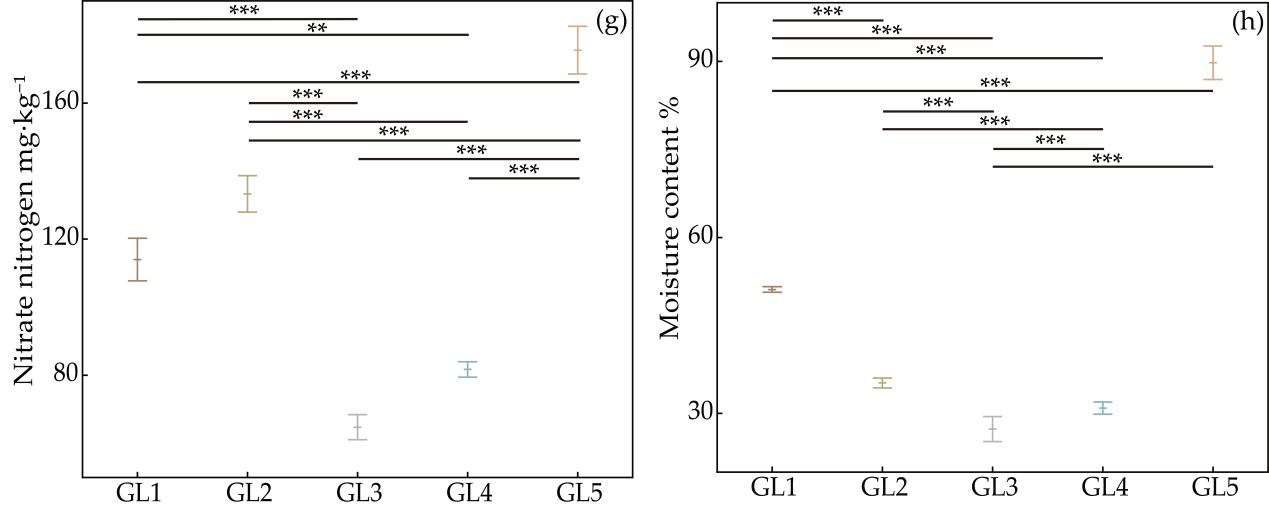


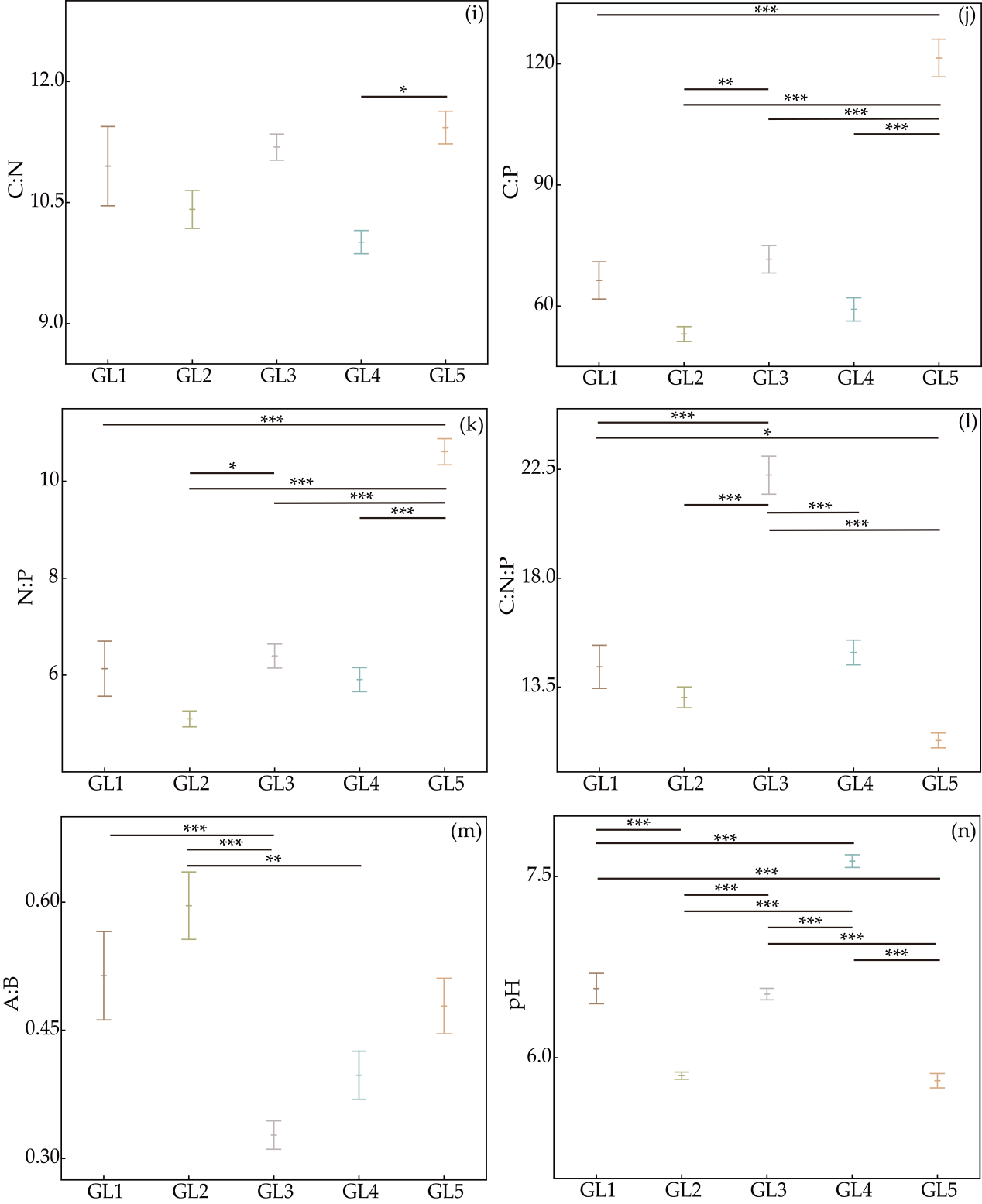
Figure S7 Ecosystem functions in different grassland types (a. Total nitrogen, b.Organic carbon, c. Total phosphorus, d. Alkali hydrolyzed nitrogen, e. Available phosphorous, f. Ammonium nitrogen, g. Nitrate nitrogen, h. Moisture content, i. Ratio of organic carbon to total nitrogen,gl. Ratio of organic carbon to total phosphorus, k. Ratio of total nitrogen to total phosphorus, l. Ratio of organic carbon to total nitrogen to total phosphorus, m. Ratio of plant aboveground biomass to belowground biomass, n. pH). *, **, and *** indicate a significant relationship with Pearson test at *P* < 0.05, *P* < 0.01, *P* < 0.001 level respectively.


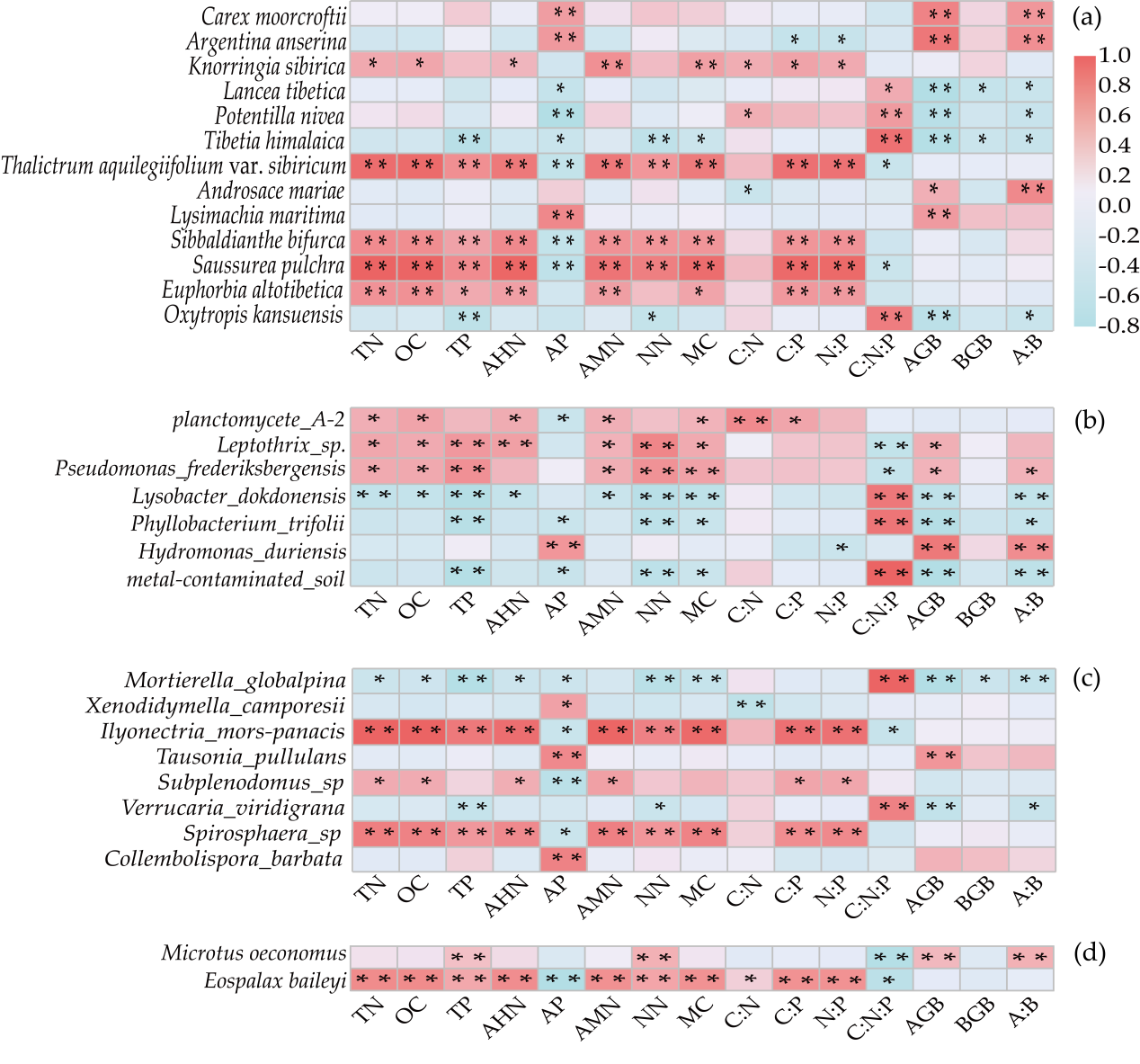


Figure S8 Pearson correlation between keystone species and ecosystem functions (a. keystone species of plant, b. keystone species of bacteria, c. keystone species of fungi, d. keystone species of rodent) and soil functions. *, **, indicate a significant relationship with Pearson test at *P* < 0.05, *P* < 0.001 level respectively. Red and blue color indicate a positive and negative relationship. “TN”, “OC”, “TP” “AHN”, “AP”, “AMN”, “NN”, “MC”, “C:N”, “C:P”, “N:P”, “C:N:P”, “AGB”, “BGB”, “A:B” represent “Total nitrogen g·kg^-1^”, “Organic carbon g·kg^-1^”, “Total phosphorus g·kg^-1^”, “Alkali hydrolyzed nitrogen mg·kg^-1^”, “Available phosphorous mg·kg^-1^”, “Ammonium nitrogen mg·kg^-1^”, “Nitrate nitrogen mg·kg^-1^”, “Moisture content %”, “Ratio of organic carbon to total nitrogen”, “Ratio of organic carbon to total phosphorus”, “Ratio of total nitrogen to total phosphorus”, “Ratio of organic carbon to total nitrogen to total phosphorus”, “plant aboveground biomass g·m^-2^”, “plant belowground biomass g·m^-2^” and “Ratio of plant aboveground biomass to belowground biomass ”respectively.


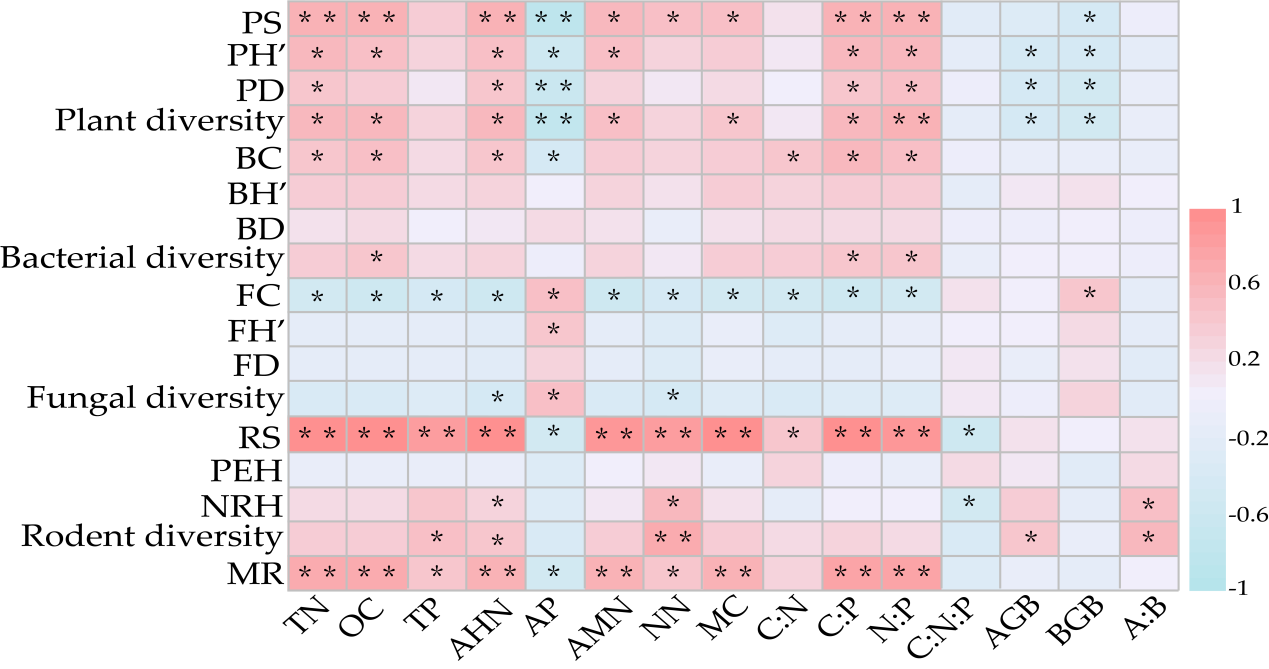


Figure S9 A visualization of a Pearson correlation matrix of different trophic diversity and ecosystem functions. *, **, indicate a significant relationship with Pearson test at *P* < 0.05, *P* < 0.001 level respectively. ”PS”, “PH”, ”PD”, ”BC”, ”BH’”, ”BD”, ”FC”, ”FH’”, ”FD”, ”RS”, ”PEH”, ”NRH”, and “MR” represent “Plant number of species”, “plant Shannon-Wiener index”, “plant Simpson”, “bacteria Chao1 index”, “bacteria Shannon-Wiener index”, “bacteria Simpson index”, “fungi Chao1 index”, “fungi Shannon-Wiener index”, “fungi Simpson index”, “rodent number of species”, ”Proportion of effective holes %”, “multitrophic diversity” and “Number of rodent holes pcs·ha^-1^” respectively. “TN”, “OC”, “TP”, “AHN”, “AP”, “AMN”, “NN”, “MC”, “C:N”, “C:P”, “N:P”, “C:N:P”, “AGB”, “BGB” and “A:B” represent “Total nitrogen g·kg^-1^”, “Organic carbon g·kg^-1^”, “Total phosphorus g·kg^-1^”, “Alkali hydrolyzed nitrogen mg·kg^-1^”, “Available phosphorous mg·kg^-1^”, “Ammonium nitrogen mg·kg^-1^”, “Nitrate nitrogen mg·kg^-1^”, “Moisture content %”, “Ratio of organic carbon to total nitrogen”, “Ratio of organic carbon to total phosphorus”, “Ratio of total nitrogen to total phosphorus”, “Ratio of organic carbon to total nitrogen to total phosphorus”, “plant aboveground biomass g·m^-2^”, “plant belowground biomass g·m^-2^” and “Ratio of plant aboveground biomass to belowground biomass” respectively.


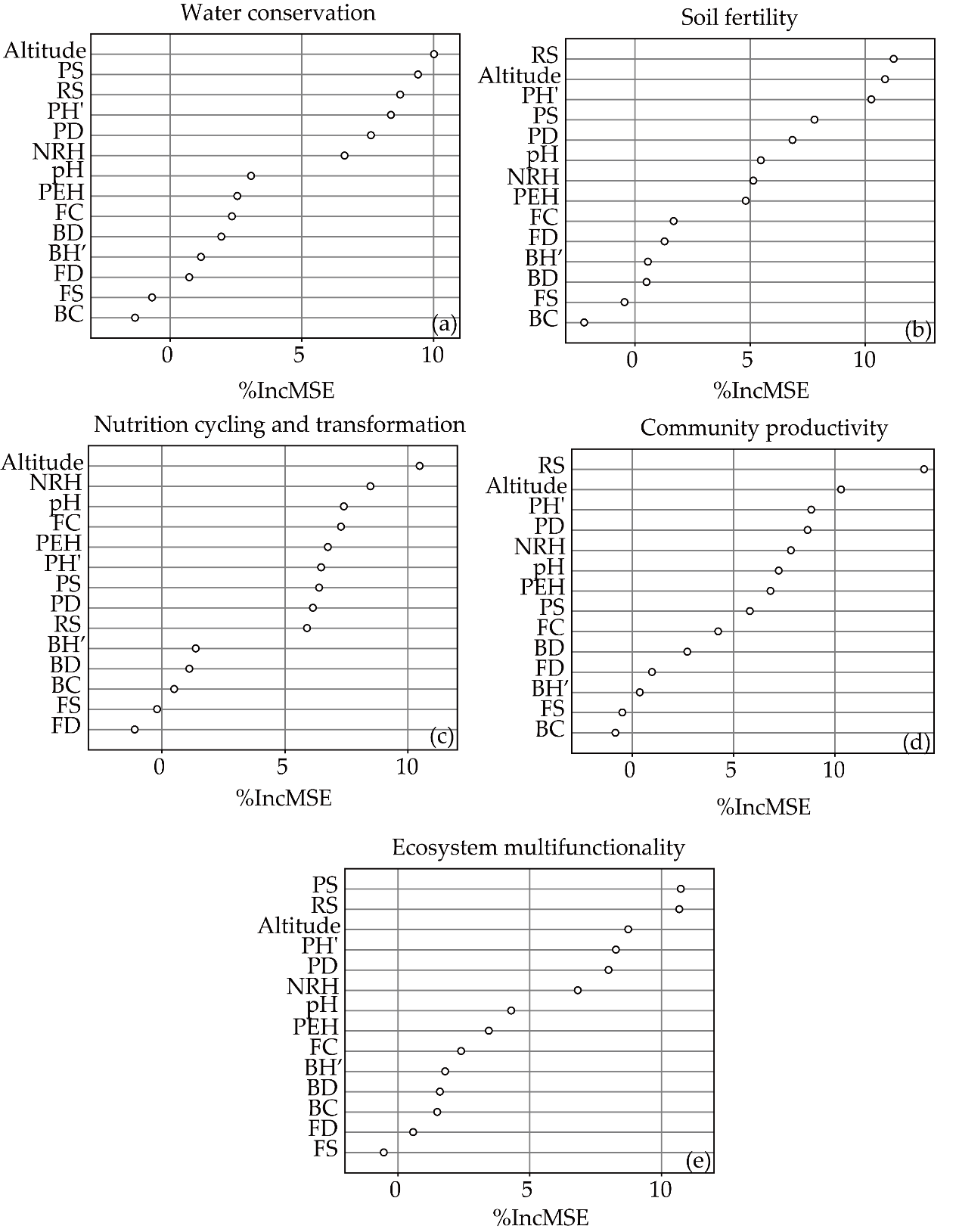


Figure S10 Results of Random Forest analysis of the relative importance of species richness of different trophic community and abiotic variables (altitude and soil pH) to ecosystem functions (a. Water conservation, b. soil fertility, c. nutrition cycling and transformation, d. community productivity, e. ecosystem multifunctionality). ”PS”, “PH”, ”PD”, ”BC”, ”BH’”, ”BD”, ”FC”, ”FH’”, ”FD”, ”RS”, ”PEH”, ”NRH” represent “Plant number of species”, “plant Shannon-Wiener index”, “plant Simpson”, “bacteria Chao1 index”, “bacteria Shannon-Wiener index”, “bacteria Simpson index”, “fungi Chao1 index”, “fungi Shannon-Wiener index”, “fungi Simpson index”, “rodent number of species”, ”Proportion of effective holes %” and “Number of rodent holes pcs·ha^-1^” respectively.
